# Symptom severity and trajectories among adolescent and young adult patients with cancer

**DOI:** 10.1093/jncics/pkad049

**Published:** 2023-11-07

**Authors:** Andrew Harper, Nicole Maseja, Reilly Parkinson, Mohammadreza Pakseresht, Sarah McKillop, Jan-Willem Henning, Linda Watson, Colleen Cuthbert, Winson Cheung, Miranda M Fidler-Benaoudia

**Affiliations:** Cancer Epidemiology and Prevention Research, Cancer Care Alberta, Alberta Health Services, Calgary, AB, Canada; Cancer Epidemiology and Prevention Research, Cancer Care Alberta, Alberta Health Services, Calgary, AB, Canada; Department of Community Health Sciences, Cumming School of Medicine, University of Calgary, Calgary, AB, Canada; Cancer Epidemiology and Prevention Research, Cancer Care Alberta, Alberta Health Services, Calgary, AB, Canada; Surveillance and Reporting, Cancer Research and Analytics, Cancer Care Alberta, Alberta Health Services, Edmonton, AB, Canada; Department of Agricultural, Food and Nutritional Science, University of Alberta, Edmonton, AB, Canada; Division of Hematology/Oncology, Stollery Children’s Hospital, Edmonton, AB, Canada; Faculty of Medicine and Dentistry, University of Alberta, Edmonton, AB, Canada; Department of Oncology, Cumming School of Medicine, University of Calgary, Calgary, AB, Canada; Faculty of Nursing, University of Calgary, Calgary, AB, Canada; Applied Research and Patient Experience, Cancer Care Alberta, Alberta Health Services, Calgary, AB, Canada; Department of Oncology, Cumming School of Medicine, University of Calgary, Calgary, AB, Canada; Faculty of Nursing, University of Calgary, Calgary, AB, Canada; Department of Oncology, Cumming School of Medicine, University of Calgary, Calgary, AB, Canada; Cancer Epidemiology and Prevention Research, Cancer Care Alberta, Alberta Health Services, Calgary, AB, Canada; Department of Community Health Sciences, Cumming School of Medicine, University of Calgary, Calgary, AB, Canada; Department of Oncology, Cumming School of Medicine, University of Calgary, Calgary, AB, Canada

## Abstract

**Background:**

Patients with cancer experience significant symptom burden. We investigated symptom severity in adolescents and young adults (18- to 39-year-olds) during the year following a cancer diagnosis and made comparisons with older adult (those older than 40 years of age) patients with cancer.

**Methods:**

All Albertan residents diagnosed with a first primary neoplasm at 18 years of age or older between April 1, 2018, and December 31, 2019, and who completed at least 1 electronic patient-reported outcome questionnaire were included. Symptom severity was assessed using the Edmonton Symptom Assessment System-revised. Descriptive statistics, multivariable logistic modeling, and mixed logistic regression modeling were used to describe symptom severity, identify risk factors, and assess symptom trajectories, respectively.

**Results:**

In total, 473 and 322 adolescents and young adults completed a patient-reported outcomes questionnaire at diagnosis and 1 year after diagnosis, respectively. Adolescent and young adult patients with cancer reported high levels of tiredness, poor well-being, and anxiety. Important risk factors included metastatic disease, female sex, treatment types received, and age at diagnosis. Symptom severity varied by clinical tumor group, with those diagnosed with sarcoma having the worst scores for all symptoms at diagnosis and patients with intrathoracic or endocrine tumors having the worst scores for all symptoms at 1 year after diagnosis. Statistically significant differences in symptom severity over the 1-year period were observed between adolescents and young adults and older adults—specifically, the odds of having moderate to severe symptoms were statistically significantly greater among adolescents and young adults with respect to pain, tiredness, nausea, depression, anxiety, and poor well-being (all *P* < .01).

**Conclusions:**

A substantial proportion of adolescents and young adults experience moderate to severe symptoms during the year following diagnosis. Modifying existing supportive services and developing interventions based on the needs of adolescent and young adult patients with cancer could aid symptom control.

Individuals diagnosed with cancer experience adverse symptoms as a result of their disease and its treatment. One systematic review of 21 multinational studies demonstrated that the following symptoms had a high prevalence across all cancer types: fatigue (59.6%), pain (48.0%), lack of appetite (45.4%), shortness of breath (43.7%), and nausea (40.1%) (1). Although prior studies have identified that symptoms vary by patient and cancer characteristics, adolescents and young adults (those 18 to 39 years of age) have largely been overlooked (1-3). This oversight is worrisome because adolescents and young adults with cancer often present with complex needs, given the unique challenges of their developmental stage, that may make them disproportionately at risk for high symptom burdens (4). Indeed, the existing literature has reported significantly higher levels of depression and anxiety as well as more concerns in the domains of “emotional, social, family, or spiritual,” and “work or school” than older adults (those 40 years of age or older) (5).

Evaluating symptoms is crucial for informing future strategies to mitigate or prevent morbidity or to improve treatment compliance, with a goal of better quality of life (6,7). As a result, patient-reported outcomes have been increasingly implemented across cancer centers as a way for clinical teams to collect direct input from patients on their well-being. Patient-reported outcomes are endorsed as a reliable mechanism to assess symptoms in a consistent manner and can lead to high-quality, patient-centered care (8,9). Though studies providing insight into patient-reported outcomes among adolescents and young adults with cancer do exist, there remains a paucity of research investigating trajectories and risk factors for high symptom burdens. Further, comparisons with older or younger cancer populations are warranted to truly understand the unique needs of this population.

Thus, we sought to address existing knowledge gaps by evaluating the well-being of adolescents and young adults diagnosed with cancer between the ages of 18 and 39 years in Alberta, Canada, through a retrospective cohort design using secondary patient-reported outcomes data. The objectives were to 1) comprehensively describe symptom severity among adolescents and young adults with cancer at diagnosis and 1 year after diagnosis and identify demographic and clinical risk factors for higher severity, 2) evaluate symptoms trajectories among adolescents and young adults with cancer during the year following diagnosis, and 3) compare symptom severity and trajectories with older adult patients with cancer.

## Methods

### Patient-reported outcomes in oncology in Alberta

The collection of patient-reported outcomes is the standard of care across the 17 ambulatory cancer centers in Alberta, with all patients treated at adult cancer centers routinely completing the Edmonton Symptom Assessment System-revised (ESAS-r) since 2014 (3,4,10). The ESAS-r is a valid and reliable patient-reported outcomes tool that assesses symptom severity for pain, tiredness, drowsiness, nausea, lack of appetite, shortness of breath, depression, anxiety, and overall well-being (11-15), where 0 represents the absence of symptoms and 10 represents the most severe symptoms. The ESAS-r has been used previously in cancer populations, including adolescents and young adults (5,16,17). For the purposes of this study, symptom severity was assessed continuously and dichotomously (0-3 = no or low symptoms; 4-10 = moderate to severe symptoms).

### Study population

The Alberta Cancer Registry provided data on all Albertan residents diagnosed with a first primary neoplasm from 18 years of age onwards between April 1, 2018, and November 30, 2019, who were not diagnosed with multiple cancers, except nonmelanoma skin cancer, and completed at least 1 electronic patient-reported outcomes questionnaire. Adolescents and young adults were defined as individuals aged 18 to 39 years, and older adults were defined as those aged 40 years or older at diagnosis, respectively. This study was approved by the Health Research Ethics Board of Alberta—Cancer Committee (HREBA.CC-21-0004) and the requirement for consent was waived.

### Statistical analyses

Bimonthly “windows” that created 60-day segments from the date of diagnosis to a maximum of 360 days were generated for each patient; the window corresponding to 0 to 60 days is referred to as *diagnosis,* and the window corresponding to 301 to 360 days is referred to as *1 year after diagnosis.* When a patient completed multiple questionnaires within 1 window, we used the worst reported score for each symptom.

Frequencies, means, and standard deviations were used to describe the study population at diagnosis and 1 year after diagnosis by the following explanatory factors: sex, geographical zone of residence at diagnosis, metastatic status at diagnosis, crude treatment (ie, radiation therapy [RT] only, chemotherapy only, RT and chemotherapy, neither), Charlson Comorbidity Index score (18), cancer stage, and clinical tumor group (Supplementary Table 1, available online). To compare with older adults, 2-sample *t* testing or χ^2^ testing was used to assess differences in descriptive characteristics between age groups. We used logistic regression modeling to assess the relationships between the dichotomized symptom severity variable and explanatory factors both at diagnosis and at 1 year after diagnosis. Mixed logistic regression modeling with an unstructured covariance structure was used to assess changes in the odds of having moderate to severe symptoms over the year following diagnosis. All multivariable models were developed by including explanatory factors associated with the dichotomized symptom severity variable in the univariable model using a 2-sided *P* < .01 to account for the assessment of multiple comparisons. All analyses were undertaken using Stata, version 17, statistical software (StataCorp LLC, College Station, TX) (19).

## Results

Of the 1787 adolescents and young adults with cancer who met our study inclusion criteria, 937 (52.4%) completed at least 1 patient-reported outcomes questionnaire in the year following diagnosis.

### Symptom severity at diagnosis

In total, 473 adolescents and young adults completed a patient-reported outcomes questionnaire at diagnosis (Table 1). The median age at diagnosis was 32.1 years, and 52% of patients were female. The most common tumor groups were hematologic (25.2%), breast (18.8%), genitourinary (15.0%), and gastrointestinal (13.3%). The majority of patients lived in Calgary (60.9%), did not have metastatic disease (55.0%), and received chemotherapy only (38.5%) or chemotherapy and RT (35.1%).

**Table 1.** Study characteristics at diagnosis and 1 year after diagnosis, by age group, with significance testing for differences by age group

Population	Diagnosis			1 y after diagnosis		
	Adolescents and young adults	Adults aged ≥40 y	Total	Adolescents and young adults	Adults aged ≥40 y	Total
No.	473	5019	5492	322	3085	3407
Age at diagnosis,^a^ y	32.12	64.18	61.42	32.19	61.78	58.98
	5.42	11.32	14.16	5.57	10.75	13.51
	2-Sample *t* test: *P* < .001			2-Sample *t* test: *P* < .001		
Sex^b^	χ^2^ test: *P* = .735			χ^2^ test: *P* = .546		
Female	247	2580	2827	191	1776	1967
	52.22	51.40	51.47	59.32	57.57	57.73
Male	226	2439	2665	131	1309	1440
	47.78	48.60	48.53	40.68	42.43	42.27
Charlson Comorbidity Index score^a^	0.13	0.57	0.53	0.13	0.41	0.38
	0.44	1.01	0.98	0.55	0.84	0.82
	2-Sample *t* test: *P* < .001			2-Sample *t* test: *P* < .001		
Diagnosis Zone^b^	χ^2^ test: *P* = .002			χ^2^ test: *P* = .067		
South	42	567	609	27	265	292
	8.88	11.30	11.09	8.39	8.59	8.57
Calgary	288	2680	2968	161	1414	1575
	60.89	53.40	54.04	50.00	45.83	46.23
Central	45	696	741	30	459	489
	9.51	13.87	13.49	9.32	14.88	14.35
Edmonton	54	704	758	68	666	734
	11.42	14.03	13.80	21.12	21.59	21.54
North	44	372	416	36	281	317
	9.30	4.71	7.57	11.18	9.11	9.30
Cancer metastasis^b^	χ^2^ test: *P* < .001			χ^2^ test: *P* < .001		
Yes	58	1415	1473	24	579	603
	12.26	28.19	26.82	7.45	18.77	17.70
No	260	2509	2769	193	1928	2121
	54.97	49.99	50.42	59.94	62.50	62.25
Missing	155	1095	1250	105	578	683
	32.77	21.82	22.76	32.61	18.74	20.05
Cancer treatment^b^	χ^2^ test: *P* < .001			χ^2^ test: *P* < .001		
Both chemotherapy and radiotherapy	166	1507	1673	135	1262	1397
	35.10	30.03	30.46	41.93	40.91	41.00
Chemotherapy only	182	1430	1612	111	983	1094
	38.48	28.49	29.35	34.47	31.86	32.11
Radiotherapy only	19	815	834	17	501	518
	4.02	16.24	15.19	5.28	16.24	15.20
Neither	106	1267	1373	59	339	398
	22.41	25.24	25.00	18.32	10.99	11.68
Cancer stage^b^	χ^2^ test: *P* < .001			χ^2^ test: *P* = .001		
I	83	824	907	85	673	758
	17.55	16.42	16.51	26.40	21.82	22.25
II	80	619	699	51	460	511
	16.91	12.33	12.73	15.84	14.91	15.00
III	81	892	973	53	715	768
	17.12	17.77	17.72	16.46	23.18	22.54
IV	89	1690	1779	50	732	782
	18.82	33.67	32.39	15.53	23.73	22.95
Missing	140	994	1134	83	505	588
	29.60	19.80	20.65	25.78	16.37	17.26
Cancer site^b^	χ^2^ test: *P* < .001			χ^2^ test: *P* < .001		
Breast	89	472	561	88	815	903
	18.82	9.40	10.21	27.33	26.42	26.50
Central nervous system	22	127	149	14	45	59
	4.65	2.53	2.71	4.35	1.46	1.73
Endocrine	3	19	22	17	37	54
	0.63	0.38	0.40	5.28	1.20	1.58
Gastrointestinal	63	1083	1146	30	597	627
	13.32	21.58	20.87	9.32	19.35	18.40
Genitourinary	71	491	562	31	315	346
	15.01	9.78	10.23	9.63	10.21	10.16
Gynecologic	40	642	682	24	214	238
	8.46	12.79	12.42	7.45	6.94	6.99
Head and neck	15	279	294	6	108	114
	3.17	5.56	5.35	1.86	3.50	3.35
Hematologic	119	776	895	84	476	560
	25.16	15.46	16.30	26.09	15.43	16.44
Intrathoracic	7	864	871	9	356	365
	1.48	17.21	15.86	2.80	11.54	10.71
Melanoma	21	156	177	13	79	92
	4.44	3.11	3.22	4.04	2.56	2.70
Sarcoma	20	73	93	6	37	43
	4.23	1.45	1.69	1.86	1.20	1.26
Other	3	37	40	0	6	6
	0.63	0.74	0.73	0.00	0.19	0.18

a Reported data: top = mean, bottom = SD.

b Reported data: top = frequency, bottom = column percentage.

**Figure 1.**
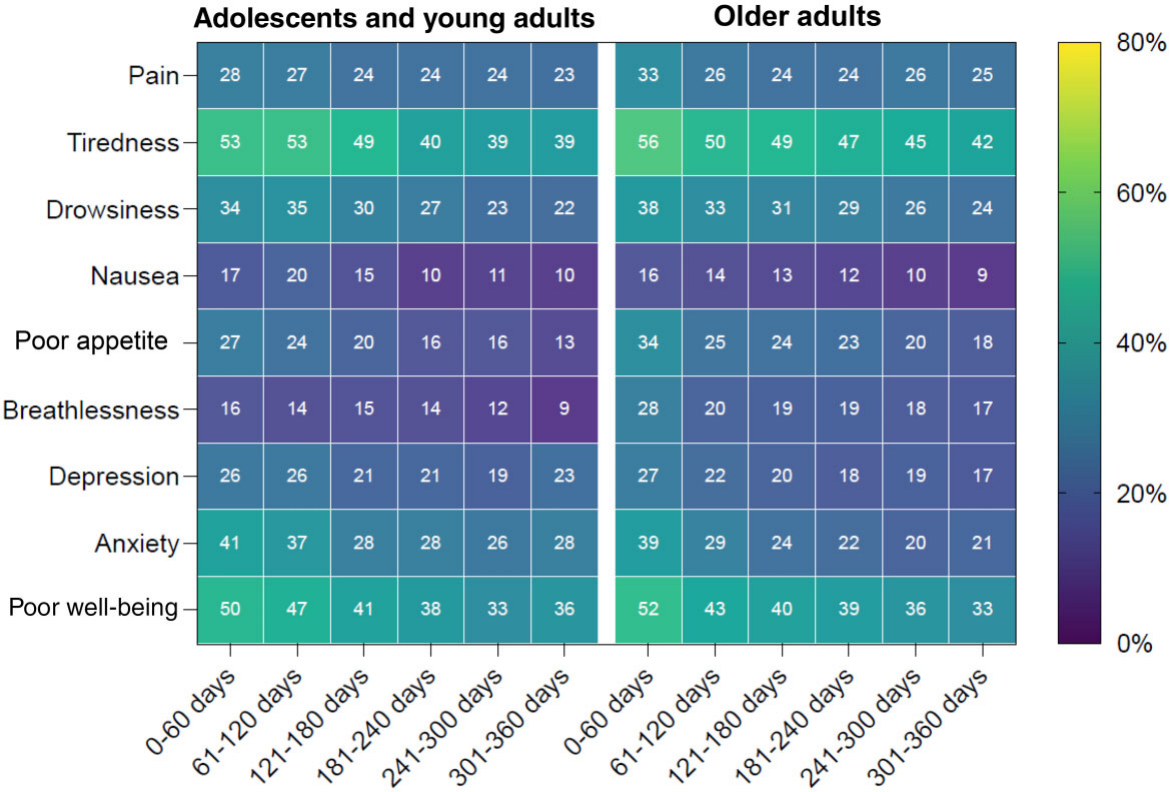
Proportion of adolescents and young adults and of older adults reporting a high symptom burden for each domain over the year following cancer diagnosis.

**Figure 2.**
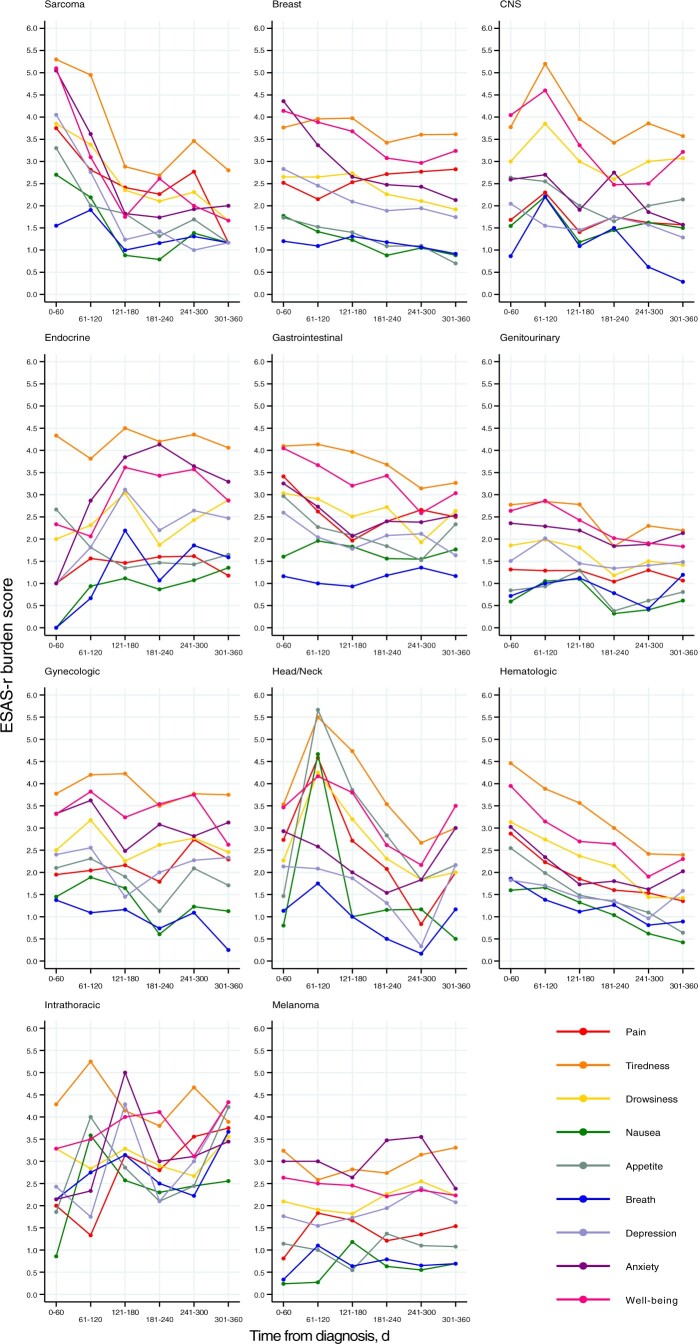
Symptom burden trajectories for each domain during the first year following cancer diagnosis among adolescents and young adults, by tumor group. CNS = central nervous system.

The proportion of adolescents and young adults with cancer reporting moderate to severe symptoms is shown in Figure 1 and Supplementary Table 2 (available online). In total, 53% of adolescents and young adults reported moderate to severe tiredness, followed by 50% reporting poor well-being, 41% reporting anxiety, and 34% reporting drowsiness. By tumor group, tiredness and poor well-being were among the top 3 symptoms for all groups (Figure 2). Notably, adolescents and young adults with sarcoma had the worst scores at diagnosis for all symptoms.

Identified risk factors for moderate to severe symptoms at diagnosis included metastatic disease for drowsiness (*P* = .004) and female sex for anxiety (*P* = .002) (Table 2). Metastatic disease was also a risk factor for poor appetite (odds ratio [OR] = 2.7; 95% confidence interval [CI] = 1.5 to 5.1; *P* = .002) after adjusting for the Charlson Comorbidity Index score, as was treatment type for poor well-being (*P* = .002) after adjusting for metastatic disease (Supplementary Table 3, available online).

**Table 2.** Summary of univariate logistic regression modeling results at diagnosis^a^

Covariate	Pain	Tiredness	Drowsiness	Nausea	Appetite	Breath	Depression	Anxiety	Well-being
Age at diagnosis,^b^ y	1.037	0.982	0.969	0.989	0.960	1.021	1.033	1.033	1.005
	0.997 to 1.078	0.950 to 1.016	0.936 to 1.004	0.947 to 1.033	0.921 to 0.992	0.973 to 1.071	0.993 to 1.075	0.998 to 1.070	0.972 to 1.040
	.069	.299	.079	.629	.016	.394	.106	.066	.755
Sex^c^ (referent = female)	0.757	0.108	0.504	0.033	0.845	0.991	0.164	0.002	0.050
Male	0.938	0.743	0.878	0.585	0.960	1.003	0.743	0.559	0.694
	0.626 to 1.407	0.517 to 1.068	0.599 to 1.287	0.358 to 0.958	0.639 to 1.443	0.609 to 1.653	0.489 to 1.129	0.385 to 0.812	0.481 to 1.001
	.757	.108	.504	.033	.845	.991	.164	.002	.050
Zone^c^ (referent = Calgary)	0.279	0.900	0.871	0.642	0.710	0.451	0.801	0.358	0.845
South	1.217	0.918	1.043	1.209	1.382	0.581	1.217	1.919	1.051
	0.602 to 2.461	0.480 to 1.755	0.524 to 2.074	0.546 to 2.678	0.691 to 2.763	0.198 to 1.710	0.592 to 2.503	1.000 to 3.686	0.542 to 2.037
	.585	.796	.904	.639	.361	.325	.594	.050	.884
Central	1.267	0.798	1.391	0.817	0.691	1.194	1.374	1.205	1.208
	0.638 to 2.515	0.426 to 1.497	0.729 to 2.652	0.346 to 1.929	0.318 to 1.501	0.521 to 2.734	0.692 to 2.727	0.634 to 2.290	0.635 to 2.298
	.499	.482	.317	.644	.350	.675	.364	.569	.564
Edmonton	1.517	0.935	1.166	0.675	1.063	1.580	1.092	1.176	1.270
	0.817 to 2.817	0.520 to 1.681	0.631 to 2.153	0.289 to 1.577	0.554 to 2.037	0.770 to 3.233	0.561 to 2.128	0.653 to 2.121	0.705 to 2.288
	.187	.821	.624	.364	.855	.213	.796	.589	.427
North	0.528	0.762	1.192	0.568	1.159	0.708	0.782	0.906	0.832
	0.225 to 1.236	0.404 to 1.438	0.615 to 2.311	0.214 to 1.511	0.576 to 2.331	0.264 to 1.896	0.359 to 1.707	0.469 to 1.751	0.436 to 1.586
	.141	.401	.603	.257	.679	.492	.537	.769	.576
Cancer metastasis^c^ (referent = no)	<0.001	0.083	0.004	0.016	<0.001	0.282	0.118	0.156	0.005
Yes	3.793	1.667	2.386	2.286	3.011	1.532	1.629	1.512	2.355
	2.089 to 6.888	0.936 to 2.967	1.329 to 4.282	1.166 to 4.479	1.639 to 5.531	0.704 to 3.335	0.884 to 3.002	0.854 to 2.678	1.304 to 4.253
	<.001	.083	.004	.016	<.001	.282	.118	.156	.005
Cancer treatment^c^ (referent = neither)	0.001	0.321	0.477	0.480	0.153	0.291	0.249	0.411	0.001
Chemotherapy only	1.365	1.410	1.320	1.584	1.676	1.878	0.930	0.807	1.934
	0.754 to 2.472	0.872 to 2.280	0.783 to 2.225	0.793 to 3.165	0.943 to 2.978	0.928 to 3.801	0.527 to 1.642	0.493 to 1.322	1.167 to 3.206
	.304	.162	.298	.192	.078	.080	.804	.395	.011
Radiotherapy only	0.797	1.661	1.842	1.341	0.759	1.469	2.327	1.277	2.500
	0.212 to 3.001	0.619 to 4.461	0.675 to 5.028	0.343 to 5.241	0.202 to 2.848	0.373 to 5.790	0.843 to 6.425	0.478 to 3.407	0.904 to 6.911
	.737	.314	.233	.673	.683	.583	.103	.626	.077
Both chemotherapy and radiotherapy	2.678	1.561	1.448	1.721	1.760	1.260	1.237	1.141	2.939
	1.499 to 4.783	0.955 to 2.550	0.853 to 2.455	0.857 to 3.456	0.984 to 3.148	0.598 to 2.654	0.704 to 2.173	0.695 to 1.874	1.753 to 4.929
	.001	.075	.170	.127	.057	.543	.459	.602	<.001
Charlson Comorbidity Index score^b^	2.198	1.414	1.434	1.461	1.823	1.073	1.157	1.075	1.922
	1.376 to 3.509	0.896 to 2.231	0.936 to 2.199	0.917 to 2.329	1.168 to 2.844	0.616 to 1.867	0.737 to 1.817	0.706 to 1.637	1.152 to 3.209
	.001	.136	.098	.110	.008	.804	.526	.736	.012
Cancer stage^c^ (referent = stage I)	0.002	0.154	0.325	0.466	0.014	0.127	0.047	0.137	0.053
Stage II	0.978	0.843	0.757	1.160	0.935	1.059	0.869	0.572	0.699
	0.471 to 2.028	0.456 to 1.559	0.393 to 1.458	0.507 to 2.652	0.457 to 1.911	0.431 to 2.602	0.444 to 1.701	0.307 to 1.069	0.374 to 1.308
	.951	.586	.405	.725	.854	.901	.682	.080	.263
Stage III	1.121	0.980	0.757	0.846	0.727	1.029	0.343	0.494	0.526
	0.552 to 2.276	0.530 to 1.812	0.393 to 1.458	0.355 to 2.017	0.348 to 1.520	0.419 to 2.525	0.156 to 0.755	0.264 to 0.925	0.279 to 0.992
	.751	.948	.405	.706	.396	.951	.008	.027	.047
Stage IV	2.815	1.658	1.257	1.561	2.044	2.149	0.889	0.691	1.170
	1.465 to 5.408	0.898 to 3.062	0.680 to 2.325	0.720 to 3.382	1.065 to 3.924	0.969 to 4.767	0.463 to 1.706	0.379 to 1.261	0.630 to 2.172
	.002	.106	.466	.259	.032	.060	.723	.229	.619
Tumor site^c^ (referent = breast)	0.003	0.052	0.083	0.257	0.012	0.384	0.735	0.147	0.022
Central nervous system	0.339	1.412	1.432	0.628	2.105	0.536	1.113	0.488	0.882
	0.092 to 1.245	0.548 to 3.637	0.549 to 3.735	0.192 to 2.050	0.770 to 5.756	0.112 to 2.553	0.406 to 3.047	0.186 to 1.279	0.343 to 2.264
	.103	.474	.463	.441	.147	.433	.835	.145	.793
Endocrine	—	1.956	1.034	—	1.842	—	—	—	0.367
	Not estimable	0.171 to 22.350	0.090 to 11.881	Not estimable	0.158 to 21.420	Not estimable	Not estimable	Not estimable	0.032 to 4.209
	—	.589	.978	—	.626	—	—	—	.421
Gastrointestinal	1.832	1.187	1.398	0.598	2.173	0.910	0.976	0.683	0.980
	0.934 to 3.594	0.620 to 2.275	0.713 to 2.742	0.267 to 1.338	1.055 to 4.477	0.367 to 2.256	0.477 to 1.993	0.357 to 1.307	0.507 to 1.893
	.078	.605	.330	.211	.035	.838	.946	.250	.951
Genitourinary	0.401	0.499	0.564	0.261	0.475	0.495	0.586	0.391	0.344
	0.182 to 0.882	0.262 to 0.950	0.274 to 1.162	0.100 to 0.683	0.194 to 1.162	0.180 to 1.361	0.279 to 1.231	0.203 to 0.753	0.177 to 0.668
	.023	.034	.121	.006	.103	.173	.158	.005	.002
Gynecologic	0.716	0.978	0.785	0.599	1.397	1.136	0.795	0.699	0.595
	0.306 to 1.674	0.464 to 2.062	0.344 to 1.788	0.233 to 1.541	0.592 to 3.301	0.420 to 3.075	0.340 to 1.859	0.330 to 1.479	0.275 to 1.285
	.441	.953	.564	.288	.445	.801	.596	.349	.186
Head and neck	1.074	0.652	0.517	0.202	0.567	1.339	0.867	0.475	0.490
	0.334 to 3.449	0.214 to 1.985	0.135 to 1.976	0.025 to 1.622	0.118 to 2.732	0.334 to 5.366	0.253 to 2.974	0.147 to 1.529	0.160 to 1.500
	.904	.451	.335	.132	.479	.680	.821	.212	.211
Hematologic	0.790	1.608	1.304	0.606	1.865	1.572	0.700	0.483	0.842
	0.429 to 1.455	0.921 to 2.806	0.732 to 2.321	0.310 to 1.183	0.990 to 3.516	0.770 to 3.210	0.374 to 1.311	0.276 to 0.846	0.480 to 1.476
	.449	.095	.367	.142	.054	.214	.265	.011	.547
Intrathoracic	0.358	1.304	2.759	0.471	0.614	0.893	0.954	0.142	0.294
	0.041 to 3.122	0.276 to 6.164	0.579 to 13.144	0.054 to 4.124	0.070 to 5.415	0.100 to 7.998	0.174 to 5.235	0.016 to 1.232	0.054 to 1.601
	.353	.738	.203	.496	.661	.919	.957	.077	.157
Melanoma	0.107	1.076	0.487	—	0.614	0.268	0.397	0.427	0.262
	0.014 to 0.842	0.415 to 2.786	0.150 to 1.578	Not estimable	0.164 to 2.306	0.033 to 2.161	0.108 to 1.466	0.157 to 1.159	0.087 to 0.795
	.034	.881	.230	—	.470	.216	.166	.095	.018
Sarcoma	1.758	2.281	2.529	1.211	3.014	0.595	1.590	1.044	1.714
	0.652 to 4.741	0.804 to 6.473	0.943 to 6.779	0.416 to 3.524	1.091 to 8.330	0.124 to 2.856	0.582 to 4.343	0.394 to 2.767	0.601 to 4.892
	.265	.121	.065	.725	.033	.517	.366	.931	.314
Other	4.296	—	4.138	1.413	7.368	2.679	1.192	0.427	1.469
	0.373 to 49.464	Not estimable	0.360 to 47.524	0.122 to 16.327	0.634 to 85.680	0.227 to 31.583	0.104 to 13.731	0.037 to 4.882	0.128 to 16.837
	.242	—	.254	.782	.111	.434	.888	.494	.757

a Reported data: top = odds ratio estimate, middle = 95% confidence interval, bottom = *P* value. *P* values in these rows correspond to overall significance of covariates in the modeling.

b Covariate is modeled as a continuous variable.

c Covariate is modeling as a categorical variable, with the reference level indicated.

### Symptom severity at 1 year after diagnosis

In total, 322 adolescents and young adults completed a patient-reported outcomes questionnaire at 1 year after diagnosis (Table 1). Nearly 60% of respondents were female, and the most common tumor groups were breast and hematologic, accounting for more than 50% of respondents when combined. Finally, the majority of patients resided in Calgary (50.0%), were diagnosed at stage I (26.4%), and were treated with chemotherapy and RT (41.9%).

At 1 year after diagnosis, the ranking of symptoms by proportion of adolescents and young adults with moderate to severe severity was as follows: tiredness (39.3%), poor well-being (35.5%), anxiety (27.8%), depression (22.5%), pain (22.5%), drowsiness (21.9%), poor appetite (13.1%), and breathlessness (9.1%) (Figure 1; Supplementary Table 2, available online). Tiredness was the most important symptom for all tumor groups, except for patients with head and neck and intrathoracic tumors, where poor well-being was a greater concern (Figure 2). Notably, patients with intrathoracic tumors reported the greatest severity for all symptoms, except tiredness, where patients with endocrine cancers reported the worst symptoms.

Risk factors for moderate to severe symptoms at 1 year after diagnosis were identified only for pain, tiredness, drowsiness, and anxiety (Table 3; Supplementary Table 4, available online). For pain, the odds of having moderate to severe symptoms was statistically significantly greater if the patient received the diagnosis at an older age (OR = 1.1; 95% CI = 1.0 to 1.2; *P* = .008), after adjusting for treatment types received. The odds of having moderate to severe tiredness was statistically significantly lower for male patients (OR = 0.4; 95% CI = 0.2 to 0.6; *P* < .001), and treatment types received was predictive of the odds of having moderate to severe drowsiness (*P* = .009) and anxiety (*P* = .004).

**Table 3.** Summary of univariate logistic regression modeling results at 1 year after diagnosis^a^

Covariate	Pain	Tiredness	Drowsiness	Nausea	Appetite	Breath	Depression	Anxiety	Well-being
Age at diagnosis,^b^ y	1.099	1.043	1.024	1.062	1.050	1.031	1.005	1.006	1.026
	1.038 to 1.164	1.000 to 1.088	0.975 to 1.076	0.985 to 1.145	0.985 to 1.121	0.958 to 1.109	0.958 to 1.053	0.962 to 1.052	0.983 to 1.071
	.001	.050	.337	.119	.135	.420	.848	.785	.233
Sex^c^ (referent = female)	0.065	<0.001	0.214	0.705	0.983	0.735	0.688	0.695	0.168
Male	0.589	0.378	0.704	0.864	0.993	0.873	0.896	0.904	0.713
	0.335 to 1.034	0.233 to 0.613	0.405 to 1.225	0.407 to 1.836	0.513 to 1.923	0.398 to 1.916	0.524 to 1.533	0.547 to 1.496	0.441 to 1.153
	.065	<.001	.214	.705	.983	.735	.668	.695	.168
Zone^c^(referent = Calgary)	0.939	0.173	0.453	0.478	0.024	0.643	0.688	0.164	0.548
South	1.082	0.459	0.985	0.905	2.803	1.531	0.842	0.743	0.744
	0.404 to 2.898	0.175 to 1.201	0.345 to 2.812	0.192 to 4.253	0.901 to 8.734	0.402 to 5.859	0.297 to 2.388	0.263 to 2.100	0.294 to 1.882
	.875	.113	.977	.899	.075	.532	.747	.575	.532
Central	1.443	0.930	1.083	0.808	1.370	1.960	0.741	1.402	1.188
	0.587 to 3.550	0.414 to 2.088	0.407 to 2.883	0.173 to 3.777	0.362 to 5.181	0.585 to 6.562	0.264 to 2.081	0.591 to 3.323	0.508 to 2.776
	.425	.861	.873	.786	.642	.275	.570	.443	.691
Edmonton	1.114	1.607	1.715	1.984	2.969	0.988	1.361	1.994	1.262
	0.558 to 2.225	0.901 to 2.866	0.884 to 3.329	0.823 to 4.779	1.276 to 6.907	0.334 to 2.923	0.704 to 2.633	1.074 to 3.702	0.693 to 2.300
	.760	.108	.111	.127	.012	.982	.359	.029	.447
North	1.263	1.022	1.733	1.824	4.111	1.976	1.425	1.635	1.700
	0.542 to 2.943	0.487 to 2.148	0.753 to 3.991	0.606 to 5.489	1.579 to 10.701	0.649 to 6.012	0.627 to 3.242	0.746 to 3.584	0.808 to 3.576
	.589	.953	.196	.285	.004	.230	.398	.219	.162
Cancer metastasis^c^ (referent = no)	0.638	0.473	0.725	0.168	0.103	0.080	0.421	0.170	0.467
Yes	1.253	0.718	0.830	2.143	2.333	2.678	0.630	0.456	0.708
	0.489 to 3.207	0.290 to 1.774	0.293 to 2.346	0.725 to 6.338	0.843 to 6.459	0.888 to 8.077	0.205 to 1.939	0.149 to 1.399	0.280 to 1.795
	.638	.472	.725	.168	.103	.080	.421	.170	.467
Cancer treatment^c^ (referent = neither)	0.002	0.127	0.009	0.270	0.207	0.847	0.069	0.004	0.0496
Chemotherapy only	1.581	0.483	0.381	0.357	0.643	0.973	0.382	0.328	0.653
	0.589 to 4.239	0.252 to 0.924	0.174 to 0.835	0.118 to 1.085	0.226 to 1.825	0.340 to 2.780	0.182 to 0.799	0.165 to 0.652	0.322 to 1.322
	.363	.028	.016	.069	.407	.959	.011	.001	.236
Radiotherapy only	1.750	1.034	2.144	0.833	1.561	1.156	0.812	0.626	2.072
	0.387 to 7.905	0.343 to 3.123	0.708 to 6.489	0.159 to 4.354	0.357 to 6.832	0.211 to 6.327	0.251 to 2.629	0.204 to 1.920	0.687 to 6.251
	.467	.952	.177	.829	.554	.868	.729	.413	.196
Both chemotherapy and radiotherapy	4.037	0.685	0.696	0.847	1.510	0.699	0.539	0.346	1.275
	1.607 to 10.146	0.370 to 1.271	0.347 to 1.396	0.341 to 2.407	0.608 to 3.746	0.242 to 2.023	0.273 to 1.061	0.180 to 0.664	0.661 to 2.461
	.003	.230	.307	.722	.374	.509	.074	.001	.469
Charlson Comorbidity Index score^b^	0.913	1.269	1.365	0.704	0.983	1.409	1.817	1.831	1.515
	0.471 to 1.770	0.838 to 1.923	0.897 to 2.076	0.241 to 2.053	0.539 to 1.795	0.881 to 2.255	1.124 to 2.937	1.111 to 3.018	0.956 to 2.402
	.787	.261	.146	.520	.956	.153	.015	.018	.077
Cancer stage^c^ (referent = stage I)	0.599	0.030	0.083	0.448	0.225	0.613	0.191	0.034	0.298
Stage II	0.680	0.682	0.518	0.280	0.734	0.819	0.407	0.287	0.503
	0.292 to 1.582	0.335 to 1.386	0.219 to 1.224	0.060 to 1.320	0.214 to 2.521	0.196 to 3.432	0.161 to 1.030	0.120 to 0.688	0.235 to 1.079
	.371	.290	.134	.108	.624	.785	.058	.005	.078
Stage III	0.617	0.326	0.301	0.701	0.880	1.638	0.511	0.542	0.611
	0.267 to 1.427	0.155 to 0.688	0.114 to 0.795	0.229 to 2.143	0.278 to 2.782	0.499 to 5.376	0.217 to 1.207	0.255 to 1.149	0.294 to 1.268
	.259	.003	.015	.533	.827	.416	.126	.110	.186
Stage IV	0.985	0.576	0.683	0.764	2.165	1.791	0.789	0.588	0.776
	0.444 to 2.186	0.281 to 1.179	0.301 to 1.548	0.249 to 2.345	0.813 to 5.768	0.544 to 5.895	0.354 to 1.763	0.276 to 1.255	0.372 to 1.621
	.971	.131	.361	.639	.122	.338	.564	.170	.501
Tumor site^c^ (referent = breast)	0.147	0.376	0.214	0.068	0.051	0.128	0.115	0.102	0.540
Central nervous system	0.555	0.928	2.099	2.364	4.571	—	0.676	0.271	1.103
	0.143 to 2.151	0.296 to 2.905	0.626 to 7.041	0.554 to 10.088	1.135 to 18.414	Not estimable	0.138 to 3.311	0.033 to 2.208	0.351 to 3.465
	.394	.897	.230	.245	.033	—	.630	.223	.867
Endocrine	0.271	2.268	2.644	1.857	3.516	2.821	2.841	3.967	0.735
	0.058 to 1.270	0.768 to 6.695	0.883 to 7.918	0.447 to 7.723	0.902 to 13.716	0.631 to 12.618	0.944 to 8.553	1.347 to 11.683	0.231 to 2.342
	.098	.138	.082	.395	.070	.175	.063	.012	.603
Gastrointestinal	0.740	0.716	1.374	2.638	3.478	2.026	1.015	1.073	1.038
	0.293 to 1.871	0.304 to 1.687	0.525 to 3.594	0.885 to 7.859	1.106 to 10.938	0.530 to 7.740	0.359 to 2.871	0.400 to 2.881	0.440 to 2.448
	.525	.445	.518	.082	.033	.302	.978	.888	.932
Genitourinary	0.302	0.506	0.907	0.289	1.693	1.951	0.974	1.511	0.448
	0.096 to 0.946	0.209 to 1.227	0.323 to 2.543	0.035 to 2.379	0.460 to 6.235	0.512 to 7.438	0.345 to 2.748	0.595 to 3.839	0.173 to 1.159
	.040	.132	.852	.248	.429	.328	.960	.385	.098
Gynecologic	0.838	1.047	1.556	1.238	2.286	—	1.671	2.984	0.735
	0.312 to 2.255	0.421 to 2.599	0.560 to 4.323	0.308 to 4.984	0.609 to 8.579	Not estimable	0.598 to 4.671	1.153 to 7.722	0.283 to 1.909
	.727	.922	.397	.764	.221	—	.327	.024	.528
Head and neck	1.018	0.618	0.756	—	2.286	2.633	2.029	3.526	0.735
	0.176 to 5.896	0.107 to 3.560	0.083 to 6.881	Not estimable	0.233 to 22.387	0.264 to 26.315	0.343 to 12.015	0.658 to 18.910	0.127 to 4.241
	.984	.590	.804	—	.478	.410	.435	.141	.731
Hematologic	0.315	0.554	0.518	0.106	0.733	0.844	0.882	1.119	0.630
	0.145 to 0.688	0.295 to 1.041	0.223 to 1.199	0.013 to 0.854	0.223 to 2.406	0.247 to 2.880	0.408 to 1.906	0.547 to 2.291	0.330 to 1.204
	.004	.066	.125	.035	.608	.787	.750	.757	.162
Intrathoracic	1.221	0.989	3.022	4.333	9.143	10.533	8.118	2.821	2.941
	0.272 to 5.480	0.248 to 3.943	0.735 to 12.425	0.921 to 20.379	1.990 to 42.010	2.225 to 49.868	1.840 to 35.805	0.689 to 11.555	0.688 to 12.573
	.794	.988	.125	.063	.004	.003	.006	.149	.146
Melanoma	0.611	1.060	1.133	0.722	0.952	1.097	1.804	1.567	0.919
	0.157 to 2.397	0.329 to 3.420	0.282 to 4.554	0.084 to 6.222	0.108 to 8.437	0.121 to 9.926	0.496 to 6.565	0.434 to 5.655	0.277 to 3.049
	.480	.922	.860	.767	.965	.934	.371	.493	.890
Sarcoma	—	0.309	0.756	1.733	2.286	2.633	Not estimable	0.882	0.294
	Not estimable	0.033 to 2.883	0.083 to 6.881	0.182 to 16.531	0.233 to 22.387	0.264 to 26.315	—	0.093 to 8.362	0.033 to 2.630
	—	.303	.804	.633	.478	.410	—	.913	.274

a Reported data: top = odds ratio estimate, middle = 95% confidence interval, bottom = *P* value. *P* values in these rows correspond to overall significance of covariates in the modeling.

b Covariate is modeled as a continuous variable.

c Covariate is modeling as a categorical variable, with the reference level indicated.

### Symptom trajectories

Across all symptoms, the proportion of adolescents and young adults reporting moderate to severe symptoms decreased from diagnosis to 1 year after diagnosis (Figure 1). After adjustment (Table 4; Supplementary Table 5, available online), there were statistically significant improvements in severity for all symptoms over the year following diagnosis, except for pain (*P* = .548). The largest improvements were observed for anxiety, poor well-being, tiredness, and drowsiness, with odds ratios below 0.8 (95% CI = 0.7 to 0.8) and *P* < .001 noted for all 4 categories. Finally, all tumor groups in general observed improvements in symptom severity over the year following diagnosis, except patients with breast cancer, whose symptoms were relatively stable, and patients with endocrine and intrathoracic cancers, where symptoms appeared to worsen with time (Figure 2).

**Table 4.** Time-adjusted mixed logistic regression models^a^ to assess the effect of time since diagnosis on the odds of having a high symptom burden (compared with a low symptom burden) among adolescents and young adult cancer survivors

ESAS-r **domain**	Model	Time effect odds ratio (95% CI)	*P*
Pain	Time^b^ + age at diagnosis^b^ + sex^c^ + metastatic disease^c^ + cancer treatment^c^ + Charlson Comorbidity Index score^b^ + tumor site^c^	0.972 (0.886 to 1.066)	.548
Tiredness	Time^b^ + age at diagnosis^b^ + Charlson Comorbidity Index score^b^ + tumor site^c^	0.757 (0.703 to 0.816)	<.001
Drowsiness	Time^b^ + sex^c^ + cancer treatment^c^ + Charlson Comorbidity score^b^	0.764 (0.705 to 0.827)	<.001
Nausea	Time^b^ + metastatic disease^c^ + Charlson Comorbidity Index score^c^ + tumor site^c^	0.830 (0.742 to 0.929)	.001
Appetite	Time^b^ + metastatic disease^c^ + cancer treatment^c^ + Charlson Comorbidity Index score^b^ + tumor site^c^	0.834 (0.751 to 0.926)	.001
Breath	Time^b^	0.872 (0.790 to 0.962)	.006
Depression	Time^b^ + sex^c^ + Charlson Comorbidity Index score^b^	0.882 (0.807 to 0.964)	.006
Anxiety	Time^b^ + sex^c^	0.740 (0.678 to 0.808)	<.001
Well-being	Time^b^ + age at diagnosis^b^ + sex^c^ + cancer treatment^c^ + Charlson Comorbidity Index score^b^ + cancer stage^c^ + tumor site^c^	0.746 (0.677 to 0.822)	<.001

a Models built based on univariate results presented in Supplementary Table S4 (available online), where covariates with *P* < .01 were included in the multivariate model. ESAS-r = Edmonton Symptom Assessment System.

b Covariate is modeled as a continuous variable.

c Covariate is modeling as a categorical variable.

### Comparison with older adults

Differences in descriptive characteristics between adolescent and young adult respondents and older adult respondents to the patient-reported outcomes questionnaire at diagnosis and at 1 year after diagnosis were noted (Table 1). Specifically, older adults were more likely to have metastatic disease and comorbidities and to be treated with RT only. The distribution by tumor group also differed, with older adults more frequently diagnosed with gastrointestinal and intrathoracic tumors.

After adjustment, the severity of all symptoms was comparable at diagnosis between adolescents and young adults and older adults (Supplementary Tables 6 and 7, available online). At 1 year after diagnosis, adolescents and young adults had a statistically significantly greater odds of having moderate to severe depression (OR = 1.7; 95% CI = 1.2 to 2.2; *P* = .001) and anxiety (OR = 1.6; 95% CI = 1.2 to 2.1; *P* = .001) compared with older adults (Supplementary Tables 7 and 8, available online). Finally, when symptom trajectories were assessed, the odds of having moderate to severe symptoms over the year were statistically significantly higher among adolescents and young adults than among older adults for pain (*P* = .003), tiredness (*P* = .005), nausea (*P* < .001), depression (*P* < .001), anxiety (*P* < .001), and poor well-being (*P* < .001), after adjustment (Supplementary Tables 7-9, available online); indeed, the odds of having moderate to severe symptoms over the year was 1.4 times (pain, tiredness) to 2.4 times (anxiety) greater for adolescents and young adults than for older adults.

## Discussion

To our knowledge, this is 1 of the most comprehensive studies of symptoms among adolescents and young adults with cancer to date and is 1 of only 3 studies in Canada to include an older adult comparison population (5,20). We revealed tiredness, pain, and anxiety as the most prominent symptoms in adolescents and young adults and that symptom trajectories varied substantially by tumor group. As we identified key risk factors for moderate to severe symptoms, we believe that it is possible to use our findings to educate health professionals about at-risk subpopulations of patients. Further, our results will aid in the development of interventions to improve operational care and symptom management because existing supportive care resources may not be adequately tailored to meet the needs of this young cancer population.

Our results show that tiredness, poor well-being, and anxiety were the greatest concerns among adolescent and young adult patients with cancer at diagnosis and 1 year after diagnosis; these results are consistent with other studies of adolescents and young adults that reported anxiety, fear, or worry as the top concern (20) and a high prevalence of fatigue, at 71% (21). The fact that these 3 symptoms consistently remained the greatest concerns over the year suggests that these are areas where additional supportive and individualized care can be targeted for adolescent and young adult patients. Although all symptoms improved with time, minimal changes were noted for depression (25.5% to 22.5%) and pain (27.8% to 22.5%). With respect to depression, the literature suggests that adolescent and young adult patients with cancer feel socially isolated during their oncology care because they are surrounded by substantially older patients (22,23). Further, given the relative rarity of cancer at young ages, adolescents and young adults with cancer rarely encounter other young people in their local setting with a similar diagnosis (24). Thus, age-appropriate group programs that connect adolescent and young adult patients with cancer or adolescent and young adult–specific spaces within cancer centers (25) could have a positive impact on minimizing depressive symptoms (24). Similarly, interventions seeking to address pain with attention to promotion of normal life activities may be more successful among adolescents and young adults (26).

Recognizing the importance of type of cancer on symptoms (27,28), we stratified findings by clinical tumor group. Notably, adolescent and young adult patients with sarcoma reported the worst scores across all symptoms at diagnosis. These findings correspond to the study by Maggi et al. (29), which reported high fatigue and pain for patients with sarcoma—a finding that correlated to poorer quality of life and psychological outcomes. Accordingly, our results identify a critical period at the beginning of these patients’ cancer journeys that would be opportune for supportive care intervention. Although 2 adolescent and young adult nurse navigators are currently integrated into cancer care in Alberta with the purpose of helping adolescent and young adult patients with cancer understand the cancer system and available support, there may be an opportunity to prevent symptoms from reaching high levels through early recognition by front-line clinicians and the creation of automatic referrals to appropriate supportive care and rehabilitative services so that resources are integrated as soon as possible.

By 1 year after diagnosis, patients with intrathoracic and endocrine cancer reported the worst symptoms. Although our results should be interpreted with caution given our small patient population, it has been well documented that patients with lung cancer experience high levels of symptoms (30-32), which may be related to advanced stage at presentation and longer treatment duration; thus, our results are not surprising. Conversely, that we identified increased moderate to severe symptoms among patients with endocrine cancer—specifically, patients with papillary thyroid cancer (94.1% of cases)—is notable. Thyroid cancer survival rates rank among the highest of all malignancies, with this cancer often being referred to as the “good cancer” (33). This positive perception may indirectly result in a lack of interventions because the good prognosis may mistakenly be associated with a lower symptom burden (34). Our results suggest the contrary, however, with young patients with thyroid cancer reporting increasing severity across all domains, except for loss of appetite. Shifting the medical perception of thyroid cancer through education is a reasonable recommendation to ensure that symptom burdens are discussed and awareness and uptake of supportive care are increased for patients with thyroid cancer.

Important risk factors for moderate to severe symptoms include metastatic disease, female sex, treatment types received, and age at diagnosis. These results correspond to the existing literature, where patients with advanced cancer have high symptom clusters of fatigue, drowsiness, nausea, decreased appetite, and dyspnea (15). Young patients with metastatic cancer are more likely to report high levels of pain (35), and female sex is associated with high levels of psychological distress, higher burdens of nausea, and worse overall ESAS-r scores (6,15,36). Similarly, previous studies found that surgery, RT, chemotherapy, and drugs used for symptom management are associated with frequent patient reports of pain and fatigue as well as lower health-related quality of life (37,38). These identified risk factors can inform interventions to better support adolescents and young adults throughout their cancer journey. For example, it may be worth adapting the existing early palliative approach for advanced cancer care (39) to adolescents and young adult because illness comprehension and coping as well as management of symptoms could differ in this younger population facing premature mortality. As adolescent and young adult oncology is still relatively underresearched, continued investigation into the needs of adolescents and young adults is required to understand how resources, interventions, and the health-care system can be modified to improve care and outcomes.

To our knowledge, only 2 other studies have compared symptoms among adolescents and young adults with those in older or younger patients with cancer (5,20). Although our analyses did not identify differences at diagnosis, a recent study suggested higher burdens among adolescents and young adults than among older adults at diagnosis for moderate to severe anxiety (52.6% and 37.0%, respectively) (20). Our study did identify significant differences at 1 year after diagnosis and in trajectories over the year following diagnosis between the 2 groups, however. These results correspond with the existing literature, with Link et al. (5) reporting higher mean scores for anxiety, depression, and nausea among adolescents and young adults than among older adults. Although cancer disrupts life for those at all ages, the impact on career development, education, and finances is particularly worrisome for adolescents and young adults, with adolescents and young adults being 3 times more likely to identify work and school as a concern compared with older adults (5,20,40). This instability may correspond with the growing differences between adolescents and young adults and older adults in time from diagnosis in our study because cancer may be a persistent strain on their personal and professional responsibilities, whereas the impact on older adults may be more immediate.

With most reports focusing on a single cancer type, this study is among the most comprehensive because symptoms were assessed for all adolescent and young adult patients with cancer (10,21,26). Given the paucity of research on this topic, our findings substantially add to the literature because we not only describe symptoms and their trajectories over a 1-year period but also investigate important explanatory factors to identify at-risk adolescent and young adult subpopulations. Further, to contextualize whether the symptoms adolescents and young adults face are distinct from other age groups, we made comparisons with older adults. As few studies have made such a comparison to date, our findings provide novel insight into the unique needs of this young cancer population (5,20).

Despite these strengths, it is important to recognize the study’s limitations. First, although considered more relevant for clinical practice (18,41), using single-item indicators for domains and dichotomizing symptom scores result in increased measurement error and loss of information, respectively. Second, we assessed the association between 8 explanatory factors and 9 symptoms, which increases the risk of type I errors. To address this concern, we used a more stringent cutoff for statistical significance (*P *< .01), which would reduce the number of false-positive findings. Third, compared with the overall adolescent and young adult cancer population in Alberta, our study population included fewer individuals from the Edmonton zone as well as fewer patients with endocrine cancer and melanoma. Differences by zone can be explained by the fact that the patient-reported outcomes questionnaire incrementally became electronic across the province, with the Edmonton tertiary cancer center (Cross Cancer Institute) not exclusively recording patient-reported outcomes electronically until October 2019; all other cancer centers recorded patient-reported outcomes electronically during the entirety of our study. Similarly, patients with endocrine cancer and melanoma treated and followed outside the cancer center by endocrinologists and dermatologists would not be eligible to complete a patient-reported outcome questionnaire, thus explaining the differences observed. Fourth, information was not available about whether the patient-reported outcome questionnaire was completed while on or off treatment; thus, we could not assess the impact of this variable on symptom severity. Finally, although we had complete ascertainment of electronic patient-reported outcomes data for our study period, it is not mandatory to complete the patient-reported outcomes questionnaire; thus, there is potential volunteer bias.

Our study provides key insights into the unique symptom burdens that adolescent and young adult patients with cancer experience as well as risk factors that influence well-being over the year following diagnosis. These findings provide anticipatory guidance on which symptoms or subpopulations warrant increased attention and intervention—insights that can be used to educate health professionals in oncology and may lead to improved symptom control for adolescents and young adults and greater efficiency within the health-care system. Our findings can be used to counsel, educate, and empower patients so that they can advocate for the services they need.

## Supplementary Material

pkad049_Supplementary_Data

## Data Availability

The data underlying this article cannot be shared publicly as they contain semi-identifiable information that could compromise research participant privacy; however, additional summary tables of count data are available from the corresponding author upon request.
