# Supplementary material for: Symptom severity and trajectories among adolescent and young adult patients with cancer
Source: JNCI Cancer Spectr. 2023 Nov 7;7(6):pkad049. doi: 10.1093/jncics/pkad049 (PMC10634503; doi:10.1093/jncics/pkad049)
Supplement: pkad049_Supplementary_Data [file pkad049_supplementary_data.pdf]

Supplementary Material

Supplementary Table 1: Classification of clinical tumor group, as used by Cancer Care Alberta

Tumor Group	Diagnosis	ICD-O-3 Site	Morphology
**Head and Neck**	lip	C00	excluding 959, 965-976, 980-998, 9991-9999 or morphologies in sarcoma group
	tongue	C01 C02	
	parotid glands	C07	
	other major salivary glands	C08	
	tonsil	C09	
	pryriform sinus	C12	
	gum	C03	
	floor of mouth	C04	
	other and unspecified parts of mouth	C06	
	palate	C05	
	oropharynx	C10	
	nasopharynx	C11	
	hypopharynx	C13	
	other and ill-defined sites within the lip, oral cavity, and pharynx	C14	
	nasal cavities, middle ear, and accessory sinus	C30 C31	
	larynx	C32	
	other bones/joints	C410-C411	excluding 959, 965-976, 980-998, 9991-9999 or morphologies in sarcoma group
	eye	C69	excluding 872-879, 959, 965-976, 980-998, 9991-9999 or morphologies in sarcoma group
	carotid body,aortic body	C754 C755	excluding 959, 965-976, 980-998, 9991-9999 or morphologies in sarcoma group
	head/face/neck, NOS	C760	
	melanoma of Head and Neck	C00-C14,C30-C32	872-879
**Gastrointestinal**	esophagus	C15	excluding 959, 965-976, 980-998, 9991-9999 or morphologies in sarcoma group
	stomach	C16	
	small intestine, including duodenum	C17	
	colon	C18	
	rectum, rectosigmoid junction, and anus	C19 C20 C21	
	liver and intrahepatic bile ducts	C22	
	gallbladder and extrahepatic bile ducts	C23 C24	
	pancreas	C25	
	other and ill-defined sites within the digestive organs	C26	
	retroperitoneum and peritoneum	C48	
**Intrathoracic**	trachea	C33	excluding 959, 965-976, 980-998, 9991-9999 or morphologies in sarcoma group
	bronchus and lung	C34	
	thymus, heart, mediastinum	C37 C38	
	other and ill-defined sites within the respiratory system and intrathoracic organs	C39	
	thorax, Nos	C761	
**Non melanoma skin**	skin	C44	excluding 872-879, 959, 965-976, 980-998, 9991-9999 or morphologies in sarcoma group
**Melanoma**	skin	C44 + 872-879	
	eye	C69 + 872-879	
	other		872-879 excluding Gyne (c51-c58) and some H&N (c00-c14 & c30-c32) and C44 and C69

**Sarcoma**	connective tissue	C49	
	sarcoma morphologies		87103','88003','88013','88023','88033','88043','88053','88123','89103','89301','89303','89313','89351','89353','89361','89363','89633','89643','89903','89913','90403','90413','90423','90433','90443','91243','91301','91303','91403','91703','91803','92501','92503','92603','92703','93303','95403','95813','88103','88113','88133','88143','88323','88333','88403','88501','88503','88513','88523','88533','88543','88553','88573','88583','88903','88913','88953','88963','89003','89013','89023','89123','89203','89213','89333','89803','89813','90201','90203','91203','91331','91333','91813','91823','91833','91843','91853','91863','91873','91903','91923','91933','91943','92203','92213','92313','92403','92423','92433','92903','93423','95603','88063','88153','88301','88303','89603','91501','91503','92511','92513','93633','93643','93703','93713','93723','93730','94733','94900','94903','95003','95223','95613' exclude Gyne
**Breast**	breast	C50	excluding 959, 965-976, 980-998, 9991-9999 or morphologies in sarcoma group
**Gynecology**	cervix uteri	C53	excluding 959, 965-976, 980-998, 9991-9999 or morphologies in sarcoma group
	placenta	C58	
	coprus uteri	C54	
	uterus	C55	
	ovary and other uterine adnexa	C56	
	other and unspecified female genital organs	C57	
	vulva	C51	
	vaginal	C52	
	melanoma of Gyne	c51-c58	morph=872-879
	sarcoma of Gyne	c51-c58	morph=sarcoma
**Genitourinary**	penis and other male genital organs	C60 C62 C63	excluding 959, 965-976, 980-998, 9991-9999 or morphologies in sarcoma group
	prostate	C61	
	bladder	C67	
	kidney and other and unspecified urinary organs	C64-C66 C68	
**CNS**	brain	C71	excluding 959, 965-976, 980-998, 9991-9999 or morphologies in sarcoma group
	other and unspecified parts of nervous system	C70	
	spinal cord and cranial nerves	C72	
	pituitary gland, craniopharyngeal gland and pineal gland	C751 C752 C753	
**Endocrine**	thyroid	C73	excluding 959, 965-976, 980-998, 9991-9999 or morphologies in sarcoma group
	adrenal	C74	
	other endocrine	C750, C758, C759	
**Hematology**	hodgkin and non-hodgkin lymphoma, leukemia, multiple myeloma & plasma cell tumors, immunoproliferative diseases, oither hematopoietic & myeloproliferative disorders		959, 965-976, 980-998, 9991-9999
		c42	excluding morphologies in sarcoma group
**Other Malignant**	bones/joints of limb	C40	excluding 872-879, 959, 965-976, 980-998, 9991-9999 or morphologies in sarcoma group
	peripheral nerves/auton. nerv. system	C47	
	other and ill-defined sites	C762-C768	

unknown Primary	C80
lymph nodes	C77

Supplementary Table 2: Summary of Dichotomized ESAS Domain Scores by age group

Symptom	Pain				Tiredness				Drowsiness			
Age Group	AYA		40+		AYA		40+		AYA		40+	
Score	0-3	4-10	0-3	4-10	0-3	4-10	0-3	4-10	0-3	4-10	0-3	4-10
Baseline 0-60 d.	338	130	3,348	1,622	222	250	2,179	2,803	311	160	3,075	1,871
	72.22	27.78	67.36	32.64	47.03	52.97	43.74	56.26	66.03	33.97	62.17	37.83
61-120 d.	405	147	4,741	1,651	262	291	3,197	3,218	359	196	4,308	2,080
	73.37	26.63	74.17	25.83	47.38	52.62	49.84	50.16	64.68	35.32	67.44	32.56
121-180 d.	389	123	4,110	1,288	261	251	2,759	2,657	358	154	3,717	1,689
	75.98	24.02	76.14	23.86	50.98	49.02	50.94	49.06	69.92	30.08	68.76	31.24
181-240 d.	363	114	3,392	1,078	289	190	2,387	2,098	349	130	3,181	1,293
	76.10	23.90	75.88	24.12	60.33	39.67	53.22	46.78	72.86	27.14	71.10	28.90
241-300 d.	296	92	2,711	937	239	150	2,014	1,656	300	88	2,688	965
	76.29	23.71	74.31	25.69	61.44	38.56	54.88	45.12	77.32	22.68	73.58	26.42
1-Year Post 301-360 d.	245	71	2,296	751	193	125	1,784	1,283	249	70	2,303	745
	77.53	22.47	75.35	24.65	60.69	39.31	58.17	41.83	78.06	21.94	75.56	24.44

Symptom	Nausea				Appetite				Breath			
Age Group	AYA		40+		AYA		40+		AYA		40+	
Score	0-3	4-10	0-3	4-10	0-3	4-10	0-3	4-10	0-3	4-10	0-3	4-10
Baseline 0-60 d.	391	81	4,208	773	344	127	3,292	1,688	399	73	3,604	1,371
	82.84	17.16	84.48	15.52	73.04	26.96	66.10	33.90	84.53	15.47	72.44	27.56
61-120 d.	442	112	5,497	899	421	134	4,776	1,623	474	76	5,109	1,277
	79.78	20.22	85.94	14.06	75.86	24.14	74.64	25.36	86.18	13.82	80.00	20.00
121-180 d.	433	79	4,711	704	407	103	4,100	1,299	434	78	4,382	1,026
	84.57	15.43	87.00	13.00	79.80	20.20	75.94	24.06	84.77	15.23	81.03	18.97
181-240 d.	433	47	3,931	549	404	74	3,442	1,042	412	65	3,633	847
	90.21	9.79	87.75	12.25	84.52	15.48	76.76	23.24	86.37	13.63	81.09	18.91
241-300 d.	346	44	3,309	349	327	62	2,947	712	344	46	3,010	643
	88.72	11.28	90.46	9.54	84.06	15.94	80.54	19.46	88.21	11.79	82.40	17.60
1-Year Post 301-360 d.	288	32	2,779	280	278	42	2,517	539	289	29	2,530	520
	90.00	10.00	90.85	9.15	86.88	13.13	82.36	17.64	90.88	9.12	82.95	17.05

Symptom	Depression				Anxiety				Wellbeing			
Age Group	AYA		40+		AYA		40+		AYA		40+	
Score	0-3	4-10	0-3	4-10	0-3	4-10	0-3	4-10	0-3	4-10	0-3	4-10
Baseline 0-60 d.	351	120	3,611	1,349	279	192	3,027	1,927	233	229	2,338	2,483
	74.52	25.48	72.80	27.20	59.24	40.76	61.10	38.90	50.43	49.57	48.50	51.50
61-120 d.	412	142	4,993	1,399	347	207	4,515	1,875	288	257	3,566	2,700
	74.37	25.63	78.11	21.89	62.64	37.36	70.66	29.34	52.84	47.16	56.91	43.09
121-180 d.	403	108	4,319	1,065	368	144	4,092	1,305	295	208	3,200	2,095
	78.86	21.14	80.22	19.78	71.88	28.13	75.82	24.18	58.65	41.35	60.43	39.57
181-240 d.	377	101	3,653	824	346	132	3,504	964	288	178	2,696	1,688
	78.87	21.13	81.59	18.41	72.38	27.62	78.42	21.58	61.80	38.20	61.50	38.50
241-300 d.	316	74	2,973	677	286	100	2,910	745	255	123	2,294	1,272
	81.03	18.97	81.45	18.55	74.09	25.91	79.62	20.38	67.46	32.54	64.33	35.67
1-Year Post 301-360 d.	248	72	2,536	506	229	88	2,409	626	200	110	1,968	985
	77.50	22.50	83.37	16.63	72.24	27.76	79.37	20.63	64.52	35.48	66.64	33.36

[a] Reported data: frequency (top); percentage within age groups (bottom).

Supplementary Table 3: Summary of Multivariate Logistic Regression Modelling Results at Baseline (AYAs Only)

Covariate	Pain	Appetite	Wellbeing
**Cancer Metastasis[2]**<br>**(R: No)**	0.052	0.002	0.011
Yes	3.808<br>0.990 – 14.638<br>0.052	2.743<br>1.462 – 5.144<br>0.002	2.269<br>1.203 – 4.279<br>0.011
**Cancer Treatment[2]**<br>**(R: None)**	0.010	-	0.002
CT Only	0.607<br>0.195 – 1.888<br>0.389	-	1.891<br>0.988 – 3.619<br>0.055
RT Only	0.374<br>0.051 – 2.729<br>0.332	-	2.988<br>0.856 – 10.433<br>0.086
Both CT and RT	2.610<br>0.906 – 7.521<br>0.076	-	3.082<br>1.723 – 5.516<br>< 0.001
**Charlson Score[1]**	2.237<br>0.971 – 5.153<br>0.059	1.878<br>0.961 – 3.670<br>0.065	-
**Cancer Stage[2]**<br>**(R: Stage I)**	0.884	-	-
Stage II	1.121<br>0.434 – 2.895<br>0.813	-	-
Stage III	0.967<br>0.370 – 2.526<br>0.946	-	-
Stage IV	1.601<br>0.379 – 6.756<br>0.522	-	-
**Tumour Site[2]**<br>**(R: Breast)**	0.267	-	-
CNS	Not Estimable	-	-
Endocrine	Not Estimable	-	-
Gastrointestinal	1.710<br>0.679 – 4.305<br>0.255	-	-
Genitourinary	2.011<br>0.445 – 9.092<br>0.364	-	-
Gynecology	1.180<br>0.434 – 3.209<br>0.746	-	-

Covariate	Pain	Appetite	Wellbeing
Head and Neck	1.317<br>0.281 – 6.178<br>0.727	-	-
Hematology	Not Estimable	-	-
Intrathoracic	0.220<br>0.018 – 2.709<br>0.237	-	-
Melanoma	0.292<br>0.032 – 2.644<br>0.273	-	-
Sarcoma	5.347<br>0.982 – 29.126<br>0.053	-	-
Other	Not Estimable	-	-

[a] Reported data: Odds ratio estimate (top); 95% CL (middle); p-value (bottom).

[b] Indicates covariate is modelled as a continuous variable.

[c] Indicates covariate is modelling as a categorical variable, with the reference level indicated. P-values in these rows correspond to overall significance of covariates in the modelling.

Supplementary Table 4: Summary of Multivariate Logistic Regression Modelling Results at 1 year post (AYAs Only)

Covariate	Pain
**Diagnosis Age**[1]	1.086<br>1.022 – 1.154<br>0.008
**Cancer Treatment**[2]<br>**(R: None)**	0.016
CT Only	1.840<br>0.573 – 5.907<br>0.305
RT Only	1.557<br>0.304 – 7.984<br>0.595
Both CT and RT	3.931<br>1.300 – 11.892<br>0.015

[a] Reported data: Odds ratio estimate (top); 95% CL (middle); p-value (bottom).

[b] Indicates covariate is modelled as a continuous variable.

[c] Indicates covariate is modelling as a categorical variable, with the reference level indicated. P-values in these rows correspond to overall significance of covariates in the modelling.

Supplementary Table 5: Time-Adjusted Demographic-Univariate Mixed Logistic Regression Modelling Results (AYAs Only)

Covariate	Pain	Tiredness	Drowsiness	Nausea	Appetite	Breath	Depression	Anxiety	Wellbeing
	1.063	1.041	1.014	1.043	0.994	1.050	1.043	1.038	1.059
**Diagnosis Age[1]**	1.030 – 1.098	1.011 – 1.072	0.981 – 1.049	1.007 – 1.079	0.964 – 1.025	1.011 – 1.090	1.006 – 1.082	1.002 – 1.075	1.025 – 1.095
	< 0.001	0.006	0.406	0.018	0.708	0.012	0.023	0.039	0.001
**Sex[2] (R: Female)**	< 0.001	< 0.001	0.005	0.112	0.916	0.305	0.002	< 0.001	< 0.001
	0.532	0.392	0.587	0.733	0.982	0.803	0.518	0.381	0.439
Male	0.375 – 0.754	0.280 – 0.549	0.404 – 0.855	0.499 – 1.075	0.694 – 1.388	0.528 – 1.221	0.343 – 0.783	0.256 – 0.568	0.297 – 0.648
	< 0.001	< 0.001	0.005	0.112	0.916	0.305	0.002	< 0.001	< 0.001
**Zone[2] (R: Calgary)**	0.272	0.495	0.253	0.946	0.163	0.398	0.179	0.116	0.275
	1.138	1.148	1.280	1.236	1.860	0.950	1.293	1.447	1.268
South	0.597 – 2.171	0.596 – 2.212	0.613 – 2.672	0.595 – 2.565	1.011 – 3.420	0.420 – 2.149	0.596 – 2.805	0.674 – 3.110	0.615 – 2.617
	0.695	0.680	0.512	0.570	0.046	0.903	0.516	0.343	0.521
	1.473	1.320	1.986	1.051	1.262	1.605	1.549	1.692	1.727
Central	0.800 – 2.711	0.721 – 2.417	1.041 – 3.788	0.546 – 2.025	0.689 – 2.311	0.800 – 3.220	0.711 – 3.376	0.816 – 3.509	0.853 – 3.495
	0.213	0.367	0.037	0.881	0.451	0.183	0.271	0.158	0.129
	1.378	1.361	1.412	0.880	1.424	1.082	1.867	1.852	1.626
Edmonton	0.876 – 2.168	0.879 – 2.107	0.878 – 2.270	0.535 – 1.448	0.910 – 2.229	0.633 – 1.849	1.119 – 3.113	1.120 – 3.060	0.993 – 2.663
	0.166	0.167	0.155	0.615	0.122	0.774	0.017	0.016	0.053
	0.703	0.839	1.242	0.958	1.643	0.590	1.091	1.609	1.126
North	0.374 – 1.322	0.478 – 1.473	0.675 – 2.285	0.489 – 1.875	0.889 – 3.034	0.264 – 1.320	0.554 – 2.146	0.857 – 3.020	0.598 – 2.124
	0.274	0.541	0.486	0.900	0.113	0.199	0.801	0.139	0.713
**Cancer Metastasis[2] (R: No)**	0.001	0.712	0.122	0.006	0.004	0.181	0.575	0.593	0.073
	2.695	1.113	1.651	2.465	2.789	1.689	1.236	0.825	1.770
Yes	1.497 – 4.853	0.630 – 1.969	0.875 – 3.117	1.292 – 4.702	1.400 – 5.555	0.791 – 3.480	0.590 – 2.588	0.406 – 1.673	0.949 – 3.304
	0.001	0.712	0.122	0.006	0.004	0.181	0.575	0.593	0.073
**Cancer Treatment[2] (R: None)**	< 0.001	0.036	0.003	0.025	0.001	0.831	0.096	0.082	< 0.001
	1.535	0.969	1.020	1.743	1.298	1.247	0.595	0.563	1.136
CT Only	0.928 – 2.538	0.615 – 1.528	0.619 – 1.682	1.000 – 3.038	0.806 – 2.092	0.717 – 2.169	0.348 – 1.015	0.335 – 0.943	0.684 – 1.884
	0.095	0.893	0.938	0.050	0.283	0.434	0.057	0.029	0.623
	2.500	1.612	3.027	2.654	1.970	0.943	1.198	1.054	3.140
RT Only	1.196 – 5.229	0.807 – 3.220	1.353 – 6.775	1.146 – 6.150	0.919 – 4.225	0.360 – 2.475	0.497 – 2.887	0.431 – 2.577	1.394 – 7.073
	0.015	0.177	0.007	0.023	0.081	0.906	0.687	0.908	0.006
	4.245	1.619	1.875	2.221	2.369	1.202	1.001	0.905	2.580
Both CT and RT	2.616 – 6.890	1.028 – 2.550	1.118 – 3.144	1.284 – 3.842	1.484 – 3.782	0.690 – 2.092	0.587 – 1.706	0.539 – 1.520	1.544 – 4.311
	< 0.001	0.038	0.017	0.004	< 0.001	0.516	0.997	0.706	< 0.001
	1.702	1.643	1.941	1.592	1.701	1.447	1.984	1.641	1.839
**Charlson Score[1]**	1.184 – 2.444	1.160 – 2.327	1.298 – 2.904	1.153 – 2.197	1.232 – 2.348	0.922 – 2.270	1.265 – 3.110	1.065 – 2.530	1.254 – 2.698
	0.004	0.005	0.001	0.005	0.001	0.108	0.003	0.025	0.002
**Cancer Stage[2] (R: Stage I)**	0.100	0.092	0.328	0.240	0.023	0.385	0.043	0.010	0.0098
	0.886	0.572	0.609	0.820	0.771	0.897	0.555	0.441	0.451
Stage II	0.506 – 1.551	0.333 – 0.982	0.332 – 1.117	0.436 – 1.539	0.430 – 1.382	0.449 – 1.790	0.294 – 1.048	0.244 – 0.799	0.246 – 0.828
	0.672	0.043	0.109	0.536	0.383	0.757	0.069	0.007	0.010
	0.827	0.541	0.694	0.897	0.682	0.739	0.409	0.434	0.427
Stage III	0.482 – 1.420	0.312 – 0.938	0.379 – 1.272	0.493 – 1.634	0.379 – 1.228	0.366 – 1.494	0.215 – 0.781	0.237 – 0.796	0.232 – 0.785

Covariate	Pain	Tiredness	Drowsiness	Nausea	Appetite	Breath	Depression	Anxiety	Wellbeing
	0.492	0.029	0.237	0.723	0.202	0.400	0.007	0.007	0.006
	1.704	0.700	0.949	1.586	1.749	1.423	0.737	0.496	0.860
Stage IV	0.973 – 2.983	0.420 – 1.167	0.538 – 1.673	0.854 – 2.945	0.994 – 3.078	0.737 – 2.750	0.379 – 1.431	0.262 – 0.936	0.482 – 1.535
	0.062	0.172	0.857	0.144	0.053	0.293	0.367	0.030	0.609
**Tumour Site[2] (R: Breast)**	< 0.001	0.001	0.011	0.006	< 0.001	0.192	0.180	0.018	0.004
	0.356	1.381	1.517	1.935	3.664	0.740	0.772	0.367	0.875
CNS	0.137 – 0.920	0.621 – 3.071	0.557 – 4.128	0.797 – 4.700	1.760 – 7631	0.240 – 2.281	0.291 – 2.047	0.134 – 0.999	0.327 – 2.341
	0.033	0.429	0.415	0.145	0.001	0.600	0.603	0.050	0.791
	0.224	2.280	1.692	0.794	1.730	1.576	1.804	1.706	0.764
Endocrine	0.082 – 0.610	0.949 – 5.481	0.717 – 3.989	0.272 – 2.315	0.747 – 4.006	0.600 – 4.143	0.664 – 4.900	0.611 – 4.763	0.289 – 2.021
	0.003	0.065	0.230	0.672	0.201	0.356	0.247	0.308	0.588
	1.004	0.811	1.201	1.696	2.391	0.812	0.974	0.791	0.936
Gastrointestinal	0.569 – 1.772	0.449 – 1.465	0.632 – 2.284	0.907 – 3.171	1.295 – 4.413	0.389 – 1.696	0.472 – 2.010	0.400 – 1.565	0.482 – 1.778
	0.989	0.488	0.575	0.098	0.005	0.579	0.942	0.501	0.817
	0.177	0.258	0.374	0.335	0.560	0.681	0.596	0.499	0.216
Genitourinary	0.089 – 0.353	0.139 – 0.479	0.182 – 0.766	0.154 – 0.730	0.290 – 1.081	0.319 – 1.456	0.288 – 1.232	0.253 – 0.984	0.108 – 0.430
	< 0.001	< 0.001	0.007	0.006	0.084	0.322	0.162	0.045	< 0.001
	0.480	1.000	1.038	1.003	1.625	0.853	1.159	1.249	0.961
Gynecology	0.232 – 0.994	0.514 – 1.946	0.477 – 2.258	0.486 – 2.069	0.836 – 3.158	0.375 – 1.941	0.509 – 2.640	0.591 – 2.641	0.450 – 2.051
	0.048	0.999	0.925	0.994	0.152	0.705	0.725	0.560	0.918
	1.068	0.734	1.263	1.600	4.445	0.792	0.994	0.508	0.669
Head and Neck	0.524 – 2.176	0.283 – 1.904	0.431 – 3.701	0.685 – 3.738	1.670 – 11.831	0.287 – 2.188	0.333 – 2.962	0.133 – 1.942	0.237 – 1.893
	0.857	0.525	0.670	0.277	0.003	0.653	0.991	0.322	0.449
	0.461	0.685	0.853	0.748	1.298	1.106	0.545	0.449	0.482
Hematology	0.282 – 0.752	0.425 – 1.103	0.503 – 1.446	0.434 – 1.288	0.807 – 2.087	0.616 – 1.985	0.300 – 0.990	0.256 – 0.788	0.277 – 0.839
	0.002	0.119	0.554	0.294	0.282	0.737	0.046	0.005	0.010
	0.805	1.427	2.711	4.265	7.581	9.013	2.590	0.891	1.227
Intrathoracic	0.202 – 3.212	0.423 – 4.813	0.753 – 9.754	0.185 – 15.345	2.032 – 28.284	1.977 – 41.096	0.627 – 10.695	0.187 – 4.243	0.279 – 5.407
	0.759	0.567	0.127	0.026	0.003	0.005	0.188	0.885	0.787
	0.227	0.700	0.955	0.294	1.047	0.740	1.280	2.115	0.383
Melanoma	0.087 – 0.593	0.269 – 1.820	0.314 – 2.899	0.091 – 0.951	0.369 – 2.974	0.215 – 2.553	0.485 – 3.374	0.771 – 5.806	0.130 – 1.126
	0.002	0.465	0.935	0.041	0.931	0.634	0.618	0.146	0.081
	0.922	1.048	1.745	1.638	2.380	0.659	0.962	0.803	0.579
Sarcoma	0.380 – 2.240	0.416 – 2.639	0.618 – 4.925	0.670 – 4.006	0.885 – 6.399	0.177 – 2.454	0.298 – 3.104	0.276 – 2.336	0.190 – 1.763
	0.859	0.920	0.293	0.279	0.086	0.534	0.948	0.688	0.336
	11.443		42.645	9.001	25.647	15.296	10.713	2.667	3.685
Other	0.321 – 408.021	Not Estimable	2.523 – 720.782	0.358 – 226.008	0.881 – 746.491	0.426 – 549.128	0.434 – 264.193	0.132 – 53.799	0.069 – 197.239
	0.181		0.009	0.182	0.059	0.135	0.147	0.522	0.521

[a] Reported data: Odds ratio estimate (top); 95% CL (middle); p-value (bottom).

[b] Indicates covariate is modelled as a continuous variable.

[c] Indicates covariate is modelling as a categorical variable, with the reference level indicated. P-values in these rows correspond to overall significance of covariates in the modelling.

Supplementary Table 6: Summary of Univariate Logistic Regression Modelling Results at diagnosis comparing AYAs to 40+

Covariate	Pain	Tiredness	Drowsiness	Nausea	Appetite	Breath	Depression	Anxiety	Wellbeing
**Diagnosis Age[2]**<br>**(R: 40+)**	0.032	0.168	0.099	0.348	0.002	< 0.001	0.422	0.428	0.426
AYA	0.794<br>0.643 – 0.980<br>0.032	0.875<br>0.725 – 1.058<br>0.168	0.846<br>0.693 – 1.032<br>0.099	1.128<br>0.877 – 1.450<br>0.348	0.720<br>0.583 – 0.890<br>0.002	0.481<br>0.372 – 0.622<br><0.001	0.915<br>0.737 – 1.136<br>0.422	1.081<br>0.892 – 1.310<br>0.428	0.925<br>0.765 – 1.120<br>0.426
				**Age-Adjusted Univariate Effects**					
**Sex[2]**<br>**(R: Female)**	0.324	0.005	0.491	< 0.001	0.402	< 0.001	0.095	< 0.001	< 0.001
Male	1.059<br>0.945 – 1.187<br>0.324	0.858<br>0.771 – 0.955<br>0.005	1.039<br>0.931 – 1.160<br>0.490	0.738<br>0.637 – 0.856<br><0.001	0.953<br>0.851 – 1.067<br>0.402	1.251<br>1.109 – 1.412<br><0.001	0.903<br>0.801 – 1.018<br>0.095	0.671<br>0.601 – 0.749<br><0.001	0.810<br>0.727 – 0.903<br><0.001
**Zone[2]**<br>**(R: Calgary)**	0.022	0.743	0.305	0.624	0.296	0.004	0.759	0.214	0.731
South	1.098<br>0.911 – 1.323<br>0.327	1.095<br>0.917 – 1.308<br>0.314	0.996<br>0.830 – 1.195<br>0.966	0.916<br>0.716 – 1.173<br>0.486	1.066<br>0.885 – 1.283<br>0.501	1.142<br>0.936 – 1.392<br>0.190	1.066<br>0.877 – 1.295<br>0.522	0.919<br>0.768 – 1.100<br>0.357	1.003<br>0.840 – 1.199<br>0.972
Central	0.930<br>0.779 – 1.111<br>0.424	0.985<br>0.837 – 1.159<br>0.856	1.121<br>0.948 – 1.325<br>0.182	1.024<br>0.821 – 1.278<br>0.831	1.031<br>0.868 – 1.226<br>0.727	1.395<br>1.167 – 1.666<br><0.001	0.905<br>0.752 – 1.090<br>0.294	0.828<br>0.699 – 0.980<br>0.028	1.049<br>0.890 – 1.237<br>0.567
Edmonton	1.242<br>1.050 – 1.470<br>0.011	1.059<br>0.901 – 1.245<br>0.483	1.029<br>0.872 – 1.214<br>0.738	0.924<br>0.738 – 1.157<br>0.492	1.197<br>1.013 – 1.415<br>0.035	1.214<br>1.015 – 1.453<br>0.034	0.973<br>0.812 – 1.166<br>0.768	0.900<br>0.763 – 1.061<br>0.208	1.108<br>0.942 – 1.304<br>0.213
North	1.239<br>0.998 – 1.537<br>0.052	1.091<br>0.886 – 1.343<br>0.414	1.221<br>0.990 – 1.507<br>0.063	1.164<br>0.888 – 1.525<br>0.271	1.113<br>0.896 – 1.382<br>0.335	1.135<br>0.899 – 1.435<br>0.287	0.986<br>0.781 – 1.244<br>0.903	0.915<br>0.740 – 1.131<br>0.410	1.084<br>0.879 – 1.337<br>0.451
**Cancer Metastasis[2]**<br>**(R: No)**	< 0.001	< 0.001	< 0.001	< 0.001	< 0.001	< 0.001	< 0.001	< 0.001	< 0.001
Yes	3.116<br>2.720 – 3.570<br>< 0.001	3.465<br>3.016 – 3.980<br><0.001	3.050<br>2.667 – 3.488<br><0.001	2.552<br>2.152 – 3.027<br><0.001	3.959<br>3.451 – 4.541<br><0.001	3.286<br>2.846 – 3.794<br><0.001	2.006<br>1.744 – 2.309<br><0.001	1.558<br>1.368 – 1.775<br><0.001	2.986<br>2.606 – 3.421<br><0.001
**Cancer Treatment[2]**<br>**(R: None)**	< 0.001	< 0.001	< 0.001	< 0.001	< 0.001	< 0.001	0.034	0.008	< 0.001
CT Only	1.586<br>1.346 – 1.868<br>< 0.001	1.545<br>1.335 – 1.788<br><0.001	1.329<br>1.139 – 1.550<br><0.001	1.642<br>1.329 – 2.029<br><0.001	1.561<br>1.333 – 1.827<br><0.001	1.300<br>1.099 – 1.537<br>0.002	1.134<br>0.959 – 1.340<br>0.141	1.065<br>0.916 – 1.238<br>0.411	1.647<br>1.419 – 1.911<br><0.001
RT Only	1.861<br>1.539 – 2.251<br>< 0.001	1.346<br>1.131 – 1.602<br>0.001	1.450<br>1.209 – 1.739<br><0.001	1.363<br>1.056 – 1.759<br>0.017	1.426<br>1.183 – 1.719<br><0.001	1.509<br>1.244 – 1.832<br><0.001	1.241<br>1.020 – 1.510<br>0.031	1.094<br>0.914 – 1.308<br>0.329	1.499<br>1.255 – 1.790<br><0.001
Both CT and RT	1.950<br>1.661 – 2.290<br>< 0.001	1.469<br>1.271 – 1.697<br><0.001	1.595<br>1.371 – 1.856<br><0.001	1.694<br>1.374 – 2.089<br><0.001	1.438<br>1.229 – 1.683<br><0.001	1.158<br>0.979 – 1.370<br>0.088	1.259<br>1.069 – 1.483<br>0.006	1.276<br>1.101 – 1.479<br>0.001	1.674<br>1.445 – 1.940<br><0.001
**Charlson Score[1]**	1.246<br>1.177 – 1.319<br>< 0.001	1.377<br>1.292 – 1.468<br><0.001	1.311<br>1.237 – 1.389<br><0.001	1.208<br>1.130 – 1.292<br><0.001	1.355<br>1.279 – 1.435<br><0.001	1.338<br>1.262 – 1.418<br><0.001	1.178<br>1.111 – 1.248<br><0.001	1.084<br>1.025 – 1.145<br>0.005	1.226<br>1.156 – 1.300<br><0.001
**Cancer Stage[2]**<br>**(R: Stage I)**	< 0.001	< 0.001	< 0.001	< 0.001	< 0.001	< 0.001	< 0.001	< 0.001	< 0.001

Covariate	Pain	Tiredness	Drowsiness	Nausea	Appetite	Breath	Depression	Anxiety	Wellbeing
Stage II	1.208<br>0.936 – 1.561<br>0.147	1.109<br>0.907 – 1.356<br>0.312	1.126<br>0.891 – 1.423<br>0.321	0.998<br>0.687 – 1.451<br>0.992	1.226<br>0.941 – 1.598<br>0.132	1.251<br>0.946 – 1.655<br>0.116	0.886<br>0.694 – 1.130<br>0.330	0.908<br>0.738 – 1.118<br>0.365	1.088<br>0.885 – 1.337<br>0.426
Stage III	1.985<br>1.588 – 2.482<br>< 0.001	1.437<br>1.196 – 1.727<br><0.001	1.659<br>1.349 – 2.042<br><0.001	2.024<br>1.490 – 2.750<br><0.001	2.138<br>1.700 – 2.690<br><0.001	1.788<br>1.398 – 2.287<br><0.001	1.077<br>0.866 – 1.339<br>0.504	1.023<br>0.847 – 1.236<br>0.811	1.403<br>1.162 – 1.695<br><0.001
Stage IV	4.267<br>3.499 – 5.203<br>< 0.001	3.748<br>3.165 – 4.437<br><0.001	3.567<br>2.970 – 4.286<br><0.001	3.601<br>2.743 – 4.727<br><0.001	5.627<br>4.587 – 6.901<br><0.001	4.219<br>3.404 – 5.231<br><0.001	1.864<br>1.547 – 2.246<br><0.001	1.403<br>1.188 – 1.656<br><0.001	3.123<br>2.633 – 3.703<br><0.001
**Tumour Site[2]**<br>**(R: Breast)**	< 0.001	< 0.001	< 0.001	< 0.001	< 0.001	< 0.001	< 0.001	< 0.001	< 0.001
CNS	0.926<br>0.607 – 1.411<br>0.719	1.851<br>1.273 – 2.692<br>0.001	2.787<br>1.922 – 4.041<br>< 0.001	1.038<br>0.616 – 1.748<br>0.889	1.132<br>0.740 – 1.733<br>0.567	1.007<br>0.620 – 1.636<br>0.976	1.036<br>0.694 – 1.545<br>0.864	0.610<br>0.417 – 0.892<br>0.011	0.997<br>0.691 – 1.439<br>0.988
Endocrine	1.343<br>0.537 – 3.362<br>0.528	1.477<br>0.621 – 3.511<br>0.377	2.028<br>0.859 – 4.788<br>0.107	0.634<br>0.145 – 2.772<br>0.545	1.045<br>0.378 – 2.889<br>0.933	1.561<br>0.557 – 4.374<br>0.397	0.958<br>0.368 – 2.492<br>0.930	0.582<br>0.233 – 1.449<br>0.245	0.958<br>0.408 – 2.246<br>0.921
Gastrointestinal	2.069<br>1.652 – 2.592<br>< 0.001	1.714<br>1.395 – 2.107<br>< 0.001	1.992<br>1.600 – 2.479<br>< 0.001	1.937<br>1.463 – 2.565<br>< 0.001	3.212<br>2.544 – 4.056<br>< 0.001	1.722<br>1.326 – 2.235<br>< 0.001	1.091<br>0.870 – 1.368<br>0.450	0.876<br>0.713 – 1.077<br>0.209	1.375<br>1.117 – 1.694<br>0.003
Genitourinary	0.797<br>0.604 – 1.053<br>0.111	0.656<br>0.517 – 0.832<br>0.001	0.831<br>0.637 – 1.083<br>0.170	0.586<br>0.399 – 0.862<br>0.007	0.834<br>0.623 – 1.117<br>0.223	0.977<br>0.711 – 1.342<br>0.885	0.564<br>0.424 – 0.750<br>< 0.001	0.443<br>0.344 – 0.570<br>< 0.001	0.471<br>0.368 – 0.603<br>< 0.001
Gynecology	0.869<br>0.668 – 1.129<br>0.292	0.780<br>0.623 – 0.978<br>0.032	0.760<br>0.588 – 0.981<br>0.035	0.783<br>0.555 – 1.105<br>0.164	0.972<br>0.740 – 1.276<br>0.836	0.826<br>0.606 – 1.126<br>0.226	0.670<br>0.515 – 0.871<br>0.003	0.730<br>0.580 – 0.919<br>0.007	0.675<br>0.536 – 0.850<br>0.001
Head and Neck	1.495<br>1.098 – 2.036<br>0.011	0.881<br>0.663 – 1.171<br>0.384	1.088<br>0.798 – 1.483<br>0.593	0.723<br>0.458 – 1.139<br>0.161	1.489<br>1.078 – 2.057<br>0.016	1.272<br>0.887 – 1.826<br>0.191	0.911<br>0.660 – 1.256<br>0.569	0.891<br>0.667 – 1.190<br>0.434	0.694<br>0.519 – 0.928<br>0.014
Hematology	1.126<br>0.885 – 1.432<br>0.333	1.501<br>1.213 – 1.858<br>< 0.001	1.542<br>1.228 – 1.936<br>< 0.001	1.058<br>0.778 – 1.437<br>0.720	1.599<br>1.250 – 2.045<br>< 0.001	1.867<br>1.428 – 2.442<br>< 0.001	0.776<br>0.610 – 0.989<br>0.040	0.705<br>0.568 – 0.875<br>0.002	1.091<br>0.880 – 1.353<br>0.425
Intrathoracic	2.294<br>1.813 – 2.903<br>< 0.001	2.958<br>2.356 – 3.714<br>< 0.001	2.717<br>2.160 – 3.419<br>< 0.001	1.778<br>1.323 – 2.391<br>< 0.001	3.386<br>2.655 – 4.319<br>< 0.001	5.888<br>4.526 – 7.659<br>< 0.001	1.637<br>1.297 – 2.066<br>< 0.001	1.137<br>0.915 – 1.411<br>0.246	1.901<br>1.520 – 2.377<br>< 0.001
Melanoma	0.392<br>0.239 – 0.643<br>< 0.001	0.457<br>0.318 – 0.655<br>< 0.001	0.389<br>0.244 – 0.622<br>< 0.001	0.185<br>0.074 – 0.466<br>< 0.001	0.328<br>0.187 – 0.578<br>< 0.001	0.706<br>0.428 – 1.164<br>0.172	0.460<br>0.293 – 0.721<br>0.001	0.579<br>0.404 – 0.828<br>0.003	0.357<br>0.243 – 0.522<br>< 0.001
Sarcoma	1.772<br>1.116 – 2.814<br>0.015	1.413<br>0.906 – 2.203<br>0.127	1.640<br>1.040 – 2.586<br>0.033	1.205<br>0.658 – 2.206<br>0.545	1.785<br>1.109 – 2.873<br>0.017	0.833<br>0.444 – 1.562<br>0.569	1.113<br>0.688 – 1.801<br>0.663	0.989<br>0.634 – 1.542<br>0.961	1.163<br>0.744 – 1.816<br>0.508
Other	4.309<br>2.225 – 8.345<br>< 0.001	4.828<br>2.100 – 11.101<br>< 0.001	4.052<br>2.081 – 7.888<br>< 0.001	4.327<br>2.195 – 8.528<br>< 0.001	7.348<br>3.679 – 14.674<br>< 0.001	3.248<br>1.659 – 6.361<br>0.001	1.379<br>0.702 – 2.711<br>0.351	1.326<br>0.692 – 2.540<br>0.395	2.162<br>1.073 – 4.357<br>0.031

[a] Reported data: Odds ratio estimate (top); 95% CL (middle); p-value (bottom).

[b] Indicates covariate is modelled as a continuous variable.

[c] Indicates covariate is modelling as a categorical variable, with the reference level indicated. P-values in these rows correspond to overall significance of covariates in the modelling.

Supplementary Table 7: Effect of age at diagnosis (AYAs vs older adults) on the odds of having a high symptom burden (compared to low symptom burden)

Subset	Model	Diagnosis Age Effect Odds Ratio (95% CI)	P-Value
**Diagnosis**			
Pain	Diagnosis age[3] + Metastatic disease[3] + Cancer treatment[3] + Charlson comorbidity score[2] + Cancer stage[3] + Tumor site[3]	1.341 (0.985 – 1.825)	0.062
Tiredness	Diagnosis age[3] + Sex[3] + Metastatic disease[3] + Cancer treatment[3] + Charlson comorbidity score[2] + Cancer stage[3] + Tumor site[3]	1.192 (0.899 – 1.580)	0.223
Drowsiness	Diagnosis age[3] + Metastatic disease[3] + Cancer treatment[3] + Charlson comorbidity score[2] + Cancer stage[3] + Tumor site[3]	1.198 (0.886 – 1.621)	0.240
Nausea	Diagnosis age[3] + Sex[3] + Metastatic disease[3] + Cancer treatment[3] + Charlson comorbidity score[2] + Cancer stage[3] + Tumor site[3]	1.627 (1.125 – 2.354)	0.010
Appetite	Diagnosis age[3] + Metastatic disease[3] + Cancer treatment[3] + Charlson comorbidity score[2] + Cancer stage[3] + Tumor site[3]	0.999 (0.713 – 1.401)	0.997
Breath	Diagnosis age[3] + Sex[3] + Diagnosis zone[3] + Metastatic disease[3] + Cancer treatment[3] + Charlson comorbidity score[2] + Cancer stage[3] + Tumor site[3]	0.797 (0.536 – 1.184)	0.261
Depression	Diagnosis age[3] + Metastatic disease[3] + Charlson comorbidity score[2] + Cancer stage[3] + Tumor site[3]	1.180 (0.867 – 1.606)	0.291
Anxiety	Diagnosis age[3] + Sex[3] + Metastatic disease[3] + Cancer treatment[3] + Charlson comorbidity score[2] + Cancer stage[3] + Tumor site[3]	1.319 (1.001 – 1.737)	0.049
Wellbeing	Diagnosis age[3] + Sex[3] + Metastatic disease[3] + Cancer treatment[3] + Charlson comorbidity score[2] + Cancer stage[3] + Tumor site[3]	1.130 (0.849 – 1.503)	0.402
**1 Year Post**			
Pain	Diagnosis age[3] + Metastatic disease[3] + Cancer treatment[3] + Charlson comorbidity score[2] + Cancer stage[3] + Tumor site[3]	1.217 (0.849 – 1.744)	0.284
Tiredness	Diagnosis age[3] + Metastatic disease[3] + Cancer treatment[3] + Charlson comorbidity score[2] + Cancer stage[3] + Tumor site[3]	1.112 (0.805 – 1.537)	0.520
Drowsiness	Diagnosis age[3] + Metastatic disease[3] + Cancer treatment[3] + Charlson comorbidity score[2] + Cancer stage[3] + Tumor site[3]	1.139 (0.784 – 1.655)	0.493
Nausea	Diagnosis age[3] + Metastatic disease[3] + Cancer treatment[3] + Charlson comorbidity score[2] + Cancer stage[3] + Tumor site[3]	1.883 (1.145 – 3.096)	0.013
Appetite	Diagnosis age[3] + Sex[3] + Diagnosis zone[3] + Metastatic disease[3] + Cancer treatment[3] + Charlson comorbidity score[2] + Cancer stage[3] + Tumor site[3]	1.045 (0.657 – 1.661)	0.853
Breath	Diagnosis age[3] + Sex[3] + Metastatic disease[3] + Charlson comorbidity score[2] + Cancer stage[3] + Tumor site[3]	0.778 (0.463 – 1.310)	0.345
Depression	Diagnosis age[3] + Charlson comorbidity score[2] + Tumor site[3]	1.665 (1.243 – 2.230)	0.001
Anxiety	Diagnosis age[3] + Charlson comorbidity score[2] + Tumor site[3]	1.620 (1.233 – 2.127)	0.001
Wellbeing	Diagnosis age[3] + Metastatic disease[3] + Cancer treatment[3] + Charlson comorbidity score[2] + Cancer stage[3] + Tumor site[3]	1.372 (0.984 – 1.913)	0.062
**Trajectories**			
Pain	Diagnosis age[3] + Time[2] + Metastatic disease[3] + Cancer treatment[3] + Charlson comorbidity score[2] + Cancer stage[3] + Tumor site[3]	1.436 (1.135 – 1.817)	0.003
Tiredness	Diagnosis age[3] + Time[2] + Metastatic disease[3] + Cancer treatment[3] + Charlson comorbidity score[2] + Cancer stage[3] + Tumor site[3]	1.399 (1.107 – 1.767)	0.005
Drowsiness	Diagnosis age[3] + Time[2] + Sex[3] + Metastatic disease[3] + Cancer treatment[3] + Charlson comorbidity score[2] + Cancer stage[3] + Tumor site[3]	1.370 (1.065 – 1.763)	0.014
Nausea	Diagnosis age[3] + Time[2] + Metastatic disease[3] + Cancer treatment[3] + Charlson comorbidity score[2] + Cancer stage[3] + Tumor site[3]	1.941 (1.483 – 2.540)	< 0.001
Appetite	Diagnosis age[3] + Time[2] + Sex[3] + Metastatic disease[3] + Cancer treatment[3] + Charlson comorbidity score[2] + Cancer stage[3] + Tumor site[3]	1.068 (0.820 – 1.391)	0.626

Subset	Model	Diagnosis Age Effect Odds Ratio (95% CI)	P-Value
Breath	Diagnosis age[3] + Time[2] + Sex[3] + Metastatic disease[3] + Cancer treatment[3] + Charlson comorbidity score[2] + Cancer stage[3] + Tumor site[3]	0.872 (0.643 – 1.183)	0.379
Depression	Diagnosis age[3] + Time[2] + Metastatic disease[3] + Cancer treatment[3] + Charlson comorbidity score[2] + Cancer stage[3] + Tumor site[3]	2.008 (1.516 – 2.659)	< 0.001
Anxiety	Diagnosis age[3] + Time[2] + Sex[3] + Metastatic disease[3] + Cancer treatment[3] + Charlson comorbidity score[2] + Cancer stage[3] + Tumor site[3]	2.414 (1.852 – 3.146)	< 0.001
Wellbeing	Diagnosis age[3] + Time[2] + Sex[3] + Metastatic disease[3] + Cancer treatment[3] + Charlson comorbidity score[2] + Cancer stage[3] + Tumor site[3]	1.611 (1.271 – 2.041)	< 0.001

[a] Models built based upon univariate results presented in Tables S6-8, where covariates with a p-value <0.01 were included in the multivariate model

[b] Indicates covariate is modelled as a continuous variable.

[c] Indicates covariate is modelling as a categorical variable, with the reference level indicated. P-values in these rows correspond to overall significance of covariates in the model

Supplementary Table 8: Summary of Univariate Logistic Regression Modelling Results at 1-year post comparing AYAs to older adults

Covariate	Pain	Tiredness	Drowsiness	Nausea	Appetite	Breath	Depression	Anxiety	Wellbeing
**Diagnosis Age[2]**<br>**(R: 40+)**	0.391	0.385	0.322	0.619	0.043	< 0.001	0.008	0.003	0.451
AYA	0.886<br>0.672 – 1.168<br>0.391	0.901<br>0.711 – 1.140<br>0.385	0.869<br>0.658 – 1.147<br>0.322	1.103<br>0.750 – 1.621<br>0.619	0.706<br>0.503 – 0.989<br>0.043	0.488<br>0.329 – 0.723<br><0.001	1.455<br>1.101 – 1.924<br>0.008	1.479<br>1.139 – 1.920<br>0.003	1.099<br>0.860 – 1.404<br>0.450
				**Age-Adjusted Univariate Effects**					
**Sex[2]**<br>**(R: Female)**	0.364	0.529	0.020	0.828	< 0.001	< 0.001	0.680	0.260	0.147
Male	0.929<br>0.792 – 1.090<br>0.364	0.956<br>0.833 – 1.099<br>0.529	1.208<br>1.031 – 1.416<br>0.020	0.974<br>0.769 – 1.234<br>0.828	1.383<br>1.156 – 1.655<br><0.001	1.404<br>1.168 – 1.687<br><0.001	1.039<br>0.867 – 1.245<br>0.680	0.908<br>0.767 – 1.074<br>0.260	0.897<br>0.774 – 1.039<br>0.147
**Zone[2]**<br>**(R: Calgary)**	0.094	0.070	0.057	0.438	0.001	0.262	0.177	0.395	0.080
South	1.289<br>0.971 – 1.712<br>0.079	1.023<br>0.792 – 1.321<br>0.861	1.464<br>1.106 – 1.937<br>0.008	1.008<br>0.655 – 1.552<br>0.972	1.183<br>0.850 – 1.645<br>0.319	1.192<br>0.854 – 1.664<br>0.301	1.132<br>0.816 – 1.569<br>0.458	1.064<br>0.783 – 1.447<br>0.691	1.150<br>0.879 – 1.505<br>0.308
Central	1.027<br>0.806 – 1.308<br>0.831	1.167<br>0.949 – 1.434<br>0.143	1.128<br>0.887 – 1.434<br>0.325	0.759<br>0.517 – 1.115<br>0.160	0.875<br>0.654 – 1.171<br>0.369	1.085<br>0.822 – 1.432<br>0.566	0.881<br>0.662 – 1.173<br>0.386	1.057<br>0.821 – 1.360<br>0.669	1.050<br>0.839 – 1.313<br>0.671
Edmonton	1.161<br>0.946 – 1.426<br>0.152	1.269<br>1.062 – 1.516<br>0.009	1.219<br>0.993 – 1.496<br>0.059	1.045<br>0.774 – 1.411<br>0.772	1.373<br>1.094 – 1.723<br>0.006	1.107<br>0.871 – 1.408<br>0.406	1.091<br>0.865 – 1.377<br>0.463	1.071<br>0.862 – 1.330<br>0.536	1.307<br>1.084 – 1.576<br>0.005
North	1.372<br>1.047 – 1.800<br>0.022	1.222<br>0.957 – 1.560<br>0.109	1.224<br>0.925 – 1.620<br>0.156	1.216<br>0.823 – 1.797<br>0.326	1.652<br>1.229 – 2.221<br>0.001	1.416<br>1.038 – 1.930<br>0.028	1.362<br>1.007 – 1.841<br>0.045	1.338<br>1.008 – 1.776<br>0.044	1.148<br>0.885 – 1.487<br>0.298
**Cancer Metastasis[2]**<br>**(R: No)**	< 0.001	< 0.001	< 0.001	< 0.001	< 0.001	< 0.001	0.035	0.034	< 0.001
Yes	1.624<br>1.330 – 1.984<br><0.001	1.670<br>1.391 – 2.006<br><0.001	1.665<br>1.362 – 2.035<br><0.001	2.350<br>1.789 – 3.087<br><0.001	2.564<br>2.068 – 3.180<br><0.001	1.878<br>1.503 – 2.347<br><0.001	1.286<br>1.018 – 1.625<br>0.035	1.264<br>1.018 – 1.569<br>0.034	1.627<br>1.345 – 1.969<br><0.001
**Cancer Treatment[2]**<br>**(R: None)**	< 0.001	< 0.001	< 0.001	0.002	< 0.001	0.032	0.282	0.149	< 0.001
CT Only	1.477<br>1.088 – 2.005<br>0.012	1.181<br>0.929 – 1.500<br>0.174	1.159<br>0.867 – 1.550<br>0.320	1.676<br>1.058 – 2.652<br>0.028	1.756<br>1.215 – 2.538<br>0.003	1.232<br>0.874 – 1.736<br>0.234	0.829<br>0.611 – 1.125<br>0.229	0.964<br>0.722 – 1.288<br>0.806	1.300<br>0.999 – 1.693<br>0.051
RT Only	1.311<br>0.927 – 1.854<br>0.126	1.205<br>0.917 – 1.582<br>0.181	1.300<br>0.939 – 1.799<br>0.114	1.027<br>0.594 – 1.777<br>0.924	1.730<br>1.153 – 2.597<br>0.008	1.211<br>0.825 – 1.779<br>0.329	1.004<br>0.711 – 1.417<br>0.983	1.054<br>0.759 – 1.464<br>0.753	1.276<br>0.946 – 1.722<br>0.111
Both CT and RT	2.167<br>1.616 – 2.907<br><0.001	1.549<br>1.229 – 1.953<br><0.001	1.608<br>1.218 – 2.124<br>0.001	1.884<br>1.205 – 2.944<br>0.005	2.310<br>1.619 – 3.298<br><0.001	1.527<br>1.098 – 2.124<br>0.012	1.013<br>0.757 – 1.355<br>0.931	1.201<br>0.911 – 1.584<br>0.195	1.782<br>1.381 – 2.298<br><0.001

Covariate	Pain	Tiredness	Drowsiness	Nausea	Appetite	Breath	Depression	Anxiety	Wellbeing
	1.192	1.258	1.301	1.285	1.295	1.339	1.315	1.199	1.285
**Charlson Score[1]**	1.090 – 1.305	1.155 – 1.370	1.190 – 1.421	1.145 – 1.443	1.179 – 1.423	1.218 – 1.472	1.196 – 1.447	1.092 – 1.315	1.178 – 1.402
	<0.001	<0.001	<0.001	<0.001	<0.001	<0.001	<0.001	<0.001	<0.001
**Cancer Stage[2] (R: Stage I)**	< 0.001	< 0.001	< 0.001	< 0.001	< 0.001	< 0.001	0.115	0.129	< 0.001
	0.760	0.843	0.897	1.276	1.416	1.119	0.858	0.864	0.981
Stage II	0.572 – 1.009	0.668 – 1.064	0.679 – 1.186	0.786 – 2.072	0.995 – 2.015	0.797 – 1.571	0.632 – 1.165	0.651 – 1.145	0.765 – 1.259
	0.057	0.151	0.447	0.324	0.053	0.515	0.326	0.308	0.882
	1.088	0.993	1.070	1.975	1.827	1.537	0.840	0.897	1.022
Stage III	0.858 – 1.381	0.809 – 1.220	0.840 – 1.364	1.314 – 2.969	1.342 – 2.487	1.152 – 2.052	0.638 – 1.104	0.698 – 1.152	0.818 – 1.277
	0.487	0.948	0.583	0.001	<0.001	0.004	0.211	0.394	0.846
	1.454	1.432	1.549	3.074	3.408	2.076	1.131	1.149	1.506
Stage IV	1.155 – 1.830	1.170 – 1.755	1.228 – 1.954	2.091 – 4.521	2.552 – 4.552	1.572 – 2.742	0.872 – 1.468	0.902 – 1.462	1.213 – 1.869
	0.001	0.001	<0.001	<0.001	<0.001	<0.001	0.352	0.260	<0.001
**Tumour Site[2] (R: Breast)**	< 0.001	< 0.001	< 0.001	< 0.001	< 0.001	< 0.001	< 0.001	0.002	< 0.001
	0.844	1.580	2.361	1.622	2.643	1.211	1.170	1.199	1.512
CNS	0.447 – 1.592	0.927 – 2.693	1.354 – 4.117	0.617 – 4.262	1.372 – 5.092	0.533 – 2.749	0.591 – 2.318	0.651 – 2.211	0.875 – 2.611
	0.600	0.093	0.002	0.326	0.004	0.648	0.652	0.560	0.138
	0.711	2.068	1.835	1.390	2.153	1.460	2.276	1.603	1.225
Endocrine	0.350 – 1.445	1.179 – 3.629	1.004 – 3.351	0.478 – 4.041	1.043 – 4.445	0.637 – 3.349	1.239 – 4.182	0.875 – 2.935	0.680 – 2.205
	0.346	0.011	0.048	0.545	0.038	0.371	0.008	0.127	0.499
	1.095	1.410	1.501	2.971	3.094	1.635	1.129	0.988	1.196
Gastrointestinal	0.866 – 1.384	1.146 – 1.734	1.182 – 1.906	2.040 – 4.325	2.326 – 4.114	1.209 – 2.211	0.854 – 1.492	0.766 – 1.274	0.960 – 1.489
	0.448	0.001	0.001	< 0.001	< 0.001	0.001	0.396	0.925	0.111
	0.682	0.830	0.890	1.388	1.516	1.580	1.007	0.926	0.765
Genitourinary	0.499 – 0.930	0.640 – 1.076	0.650 – 1.219	0.833 – 2.311	1.043 – 2.203	1.101 – 2.267	0.713 – 1.422	0.678 – 1.266	0.576 – 1.016
	0.016	0.160	0.468	0.208	0.029	0.013	0.969	0.631	0.065
	0.911	0.847	0.852	1.975	1.030	0.904	1.002	0.928	0.937
Gynecology	0.650 – 1.278	0.628 – 1.141	0.590 – 1.231	1.171 – 3.331	0.640 – 1.658	0.559 – 1.462	0.675 – 1.488	0.648 – 1.327	0.685 – 1.282
	0.590	0.274	0.393	0.011	0.903	0.682	0.991	0.681	0.684
	0.881	0.893	1.458	1.815	2.815	1.268	1.225	1.116	0.741
Head and Neck	0.554 – 1.400	0.594 – 1.340	0.935 – 2.274	0.888 – 3.708	1.734 – 4.572	0.707 – 2.275	0.730 – 2.056	0.695 – 1.794	0.467 – 1.174
	0.591	0.583	0.096	0.102	< 0.001	0.426	0.442	0.649	0.202
	0.763	0.882	0.961	1.290	1.494	1.354	0.900	0.788	0.932
Hematology	0.590 – 0.987	0.708 – 1.099	0.738 – 1.253	0.825 – 2.017	1.078 – 2.071	0.978 – 1.873	0.667 – 1.214	0.599 – 1.036	0.738 – 1.177
	0.039	0.264	0.771	0.265	0.016	0.068	0.490	0.088	0.557
	1.643	2.018	2.198	4.094	4.205	6.010	2.059	1.677	1.984
Intrathoracic	1.262 – 2.139	1.576 – 2.585	1.681 – 2.876	2.739 – 6.118	3.075 – 5.749	4.457 – 8.104	1.528 – 2.774	1.269 – 2.216	1.536 – 2.562
	< 0.001	< 0.001	< 0.001	< 0.001	< 0.001	< 0.001	< 0.001	< 0.001	< 0.001
	0.685	1.107	1.205	1.263	1.380	0.933	1.040	0.894	1.056
Melanoma	0.396 – 1.186	0.716 – 1.713	0.727 – 1.998	0.524 – 3.046	0.724 – 2.633	0.454 – 1.919	0.580 – 1.864	0.521 – 1.536	0.665 – 1.674
	0.177	0.647	0.470	0.602	0.328	0.851	0.894	0.686	0.818

Covariate	Pain	Tiredness	Drowsiness	Nausea	Appetite	Breath	Depression	Anxiety	Wellbeing
	0.484	1.069	1.318	2.942	1.492	1.970	0.866	0.635	1.020
Sarcoma	0.201 – 1.161	0.569 – 2.008	0.652 – 2.665	1.180 – 7.331	0.612 – 3.635	0.886 – 4.378	0.358 – 2.095	0.263 – 1.536	0.530 – 1.962
	0.104	0.836	0.442	0.021	0.379	0.096	0.750	0.314	0.953
	0.596	0.782	0.759	3.812	4.497	1.619	2.862	0.789	Not
Other	0.069 – 5.130	0.142 – 4.292	0.088 – 6.537	0.436 – 33.319	0.812 – 24.910	0.187 – 14.011	0.519 – 15.785	0.092 – 6.795	Estimable
	0.638	0.777	0.802	0.226	0.085	0.662	0.227	0.829	

[a] Reported data: Odds ratio estimate (top); 95% CL (middle); p-value (bottom).

[b] Indicates covariate is modelled as a continuous variable.

[c] Indicates covariate is modelling as a categorical variable, with the reference level indicated. P-values in these rows correspond to overall significance of covariates in the modelling.

Supplementary Table 9: Time-Adjusted Demographic-Univariate Mixed Logistic Regression Modelling Results comparing AYAs to older adults

Covariate	Pain	Tiredness	Drowsiness	Nausea	Appetite	Breath	Depression	Anxiety	Wellbeing
**Diagnosis Age[2]**<br>**(R: 40+)**	0.160	0.114	0.302	0.014	< 0.001	< 0.001	0.018	< 0.001	0.579
AYA	0.879<br>0.735 – 1.052<br>0.160	0.872<br>0.735 – 1.034<br>0.114	0.908<br>0.756 – 1.091<br>0.302	1.280<br>1.052 – 1.557<br>0.014	0.697<br>0.580 – 0.837<br>< 0.001	0.446<br>0.357 – 0.557<br>< 0.001	1.288<br>1.044 – 1.590<br>0.018	1.685<br>1.380 – 2.058<br>< 0.001	1.052<br>0.880 – 1.258<br>0.579
				**Time-Adjusted & Age-Adjusted Univariate Effects**					
**Sex[2]**<br>**(R: Female)**	0.073	0.086	0.002	0.915	< 0.001	< 0.001	0.012	< 0.001	< 0.001
Male	1.095<br>0.991 – 1.210<br>0.073	0.920<br>0.836 – 1.012<br>0.086	1.172<br>1.060 – 1.296<br>0.002	1.006<br>0.897 – 1.129<br>0.915	1.308<br>1.182 – 1.449<br>< 0.001	1.789<br>1.590 – 2.012<br>< 0.001	0.860<br>0.765 – 0.968<br>0.012	0.620<br>0.554 – 0.693<br>< 0.001	0.837<br>0.760 – 0.922<br>< 0.001
**Zone[2]**<br>**(R: Calgary)**	0.543	0.890	0.916	0.272	0.167	0.011	0.015	0.053	0.074
South	1.066<br>0.892 – 1.273<br>0.482	1.088<br>0.919 – 1.289<br>0.327	1.070<br>0.894 – 1.281<br>0.461	1.020<br>0.834 – 1.249<br>0.844	1.177<br>0.983 – 1.409<br>0.076	1.252<br>1.020 – 1.536<br>0.031	1.000<br>0.811 – 1.232<br>0.996	0.870<br>0.713 – 1.061<br>0.169	0.972<br>0.820 – 1.153<br>0.747
Central	0.948<br>0.816 – 1.102<br>0.489	0.997<br>0.864 – 1.151<br>0.969	1.058<br>0.911 – 1.230<br>0.460	0.972<br>0.820 – 1.153<br>0.746	0.926<br>0.793 – 1.080<br>0.327	1.217<br>1.022 – 1.448<br>0.027	0.774<br>0.648 – 0.926<br>0.005	0.780<br>0.660 – 0.921<br>0.003	0.903<br>0.781 – 1.044<br>0.167
Edmonton	1.056<br>0.927 – 1.203<br>0.409	1.031<br>0.910 – 1.169<br>0.630	1.026<br>0.900 – 1.170<br>0.700	0.853<br>0.731 – 0.995<br>0.044	1.061<br>0.929 – 1.213<br>0.381	0.907<br>0.776 – 1.059<br>0.216	1.045<br>0.898 – 1.216<br>0.570	0.926<br>0.801 – 1.070<br>0.296	1.121<br>0.988 – 1.271<br>0.075
North	1.104<br>0.927 – 1.316<br>0.267	1.013<br>0.856 – 1.200<br>0.876	1.004<br>0.840 – 1.200<br>0.964	1.047<br>0.854 – 1.283<br>0.662	1.101<br>0.920 – 1.319<br>0.295	1.038<br>0.838 – 1.285<br>0.734	0.828<br>0.672 – 1.020<br>0.076	0.917<br>0.756 – 1.113<br>0.381	0.915<br>0.773 – 1.082<br>0.298
**Cancer Metastasis[2]**<br>**(R: No)**	< 0.001	< 0.001	< 0.001	< 0.001	< 0.001	< 0.001	< 0.001	< 0.001	< 0.001
Yes	3.432<br>3.007 – 3.917<br>< 0.001	3.505<br>3.063 – 4.011<br>< 0.001	3.582<br>3.112 – 4.123<br>< 0.001	3.391<br>2.921 – 3.936<br>< 0.001	5.702<br>4.936 – 6.586<br>< 0.001	5.845<br>4.955 – 6.894<br>< 0.001	2.623<br>2.245 – 3.064<br>< 0.001	1.916<br>1.655 – 2.219<br>< 0.001	3.071<br>2.694 – 3.500<br>< 0.001
**Cancer Treatment[2]**<br>**(R: None)**	< 0.001	< 0.001	< 0.001	< 0.001	< 0.001	< 0.001	< 0.001	< 0.001	< 0.001
CT Only	1.839<br>1.580 – 2.142<br>< 0.001	1.886<br>1.636 – 2.174<br>< 0.001	1.835<br>1.578 – 2.134<br>< 0.001	3.027<br>2.509 – 3.653<br>< 0.001	2.426<br>2.078 – 2.832<br>< 0.001	1.655<br>1.390 – 1.971<br>< 0.001	1.182<br>0.995 – 1.404<br>0.057	1.085<br>0.923 – 1.275<br>0.321	1.719<br>1.491 – 1.981<br>< 0.001
RT Only	1.629<br>1.387 – 1.912<br>< 0.001	1.658<br>1.429 – 1.922<br>< 0.001	1.700<br>1.451 – 1.992<br>< 0.001	1.476<br>1.196 – 1.822<br>< 0.001	1.311<br>1.108 – 1.551<br>0.002	1.106<br>0.917 – 1.334<br>0.292	1.255<br>1.046 – 1.505<br>0.014	1.108<br>0.935 – 1.314<br>0.234	1.268<br>1.093 – 1.473<br>0.002
Both CT and RT	3.014<br>2.602 – 3.491<br>< 0.001	2.398<br>2.089 – 2.752<br>< 0.001	2.473<br>2.134 – 2.865<br>< 0.001	3.381<br>2.812 – 4.065<br>< 0.001	3.021<br>2.600 – 3.510<br>< 0.001	1.769<br>1.492 – 2.097<br>< 0.001	1.495<br>1.265 – 1.767<br>< 0.001	1.477<br>1.263 – 1.727<br>< 0.001	2.245<br>1.957 – 2.576<br>< 0.001

Covariate	Pain	Tiredness	Drowsiness	Nausea	Appetite	Breath	Depression	Anxiety	Wellbeing
**Charlson Score[1]**	1.442<br>1.363 – 1.525<br>< 0.001	1.551<br>1.462 – 1.645<br>< 0.001	1.558<br>1.470 – 1.652<br>< 0.001	1.379<br>1.301 – 1.462<br>< 0.001	1.613<br>1.523 – 1.709<br>< 0.001	1.791<br>1.677 – 1.914<br>< 0.001	1.451<br>1.359 – 1.549<br>< 0.001	1.263<br>1.189 – 1.342<br>< 0.001	1.464<br>1.385 – 1.547<br>< 0.001
**Cancer Stage[2]<br>(R: Stage I)**	< 0.001	< 0.001	< 0.001	< 0.001	< 0.001	< 0.001	< 0.001	< 0.001	< 0.001
Stage II	1.187<br>1.010 – 1.394<br>0.037	0.968<br>0.835 – 1.122<br>0.666	1.015<br>0.865 – 1.190<br>0.860	1.354<br>1.105 – 1.659<br>0.003	1.384<br>1.163 – 1.648<br>< 0.001	1.380<br>1.132 – 1.682<br>0.001	0.783<br>0.646 – 0.948<br>0.012	0.833<br>0.698 – 0.994<br>0.043	0.977<br>0.839 – 1.138<br>0.765
Stage III	1.724<br>1.481 – 2.007<br>< 0.001	1.401<br>1.215 – 1.617<br>< 0.001	1.488<br>1.279 – 1.731<br>< 0.001	2.266<br>1.880 – 2.730<br>< 0.001	2.528<br>2.150 – 2.972<br>< 0.001	2.258<br>1.874 – 2.719<br>< 0.001	1.034<br>0.868 – 1.232<br>0.709	0.962<br>0.816 – 1.135<br>0.649	1.319<br>1.142 – 1.523<br>< 0.001
Stage IV	3.808<br>3.292 – 4.405<br>< 0.001	3.215<br>2.796 – 3.697<br>< 0.001	3.526<br>3.039 – 4.089<br>< 0.001	4.462<br>3.736 – 5.330<br>< 0.001	7.330<br>6.258 – 8.587<br>< 0.001	6.493<br>5.435 – 7.758<br>< 0.001	2.189<br>1.853 – 2.586<br>< 0.001	1.603<br>1.370 – 1.876<br>< 0.001	2.820<br>2.454 – 3.242<br>< 0.001
**Tumour Site[2]<br>(R: Breast)**	< 0.001	< 0.001	< 0.001	< 0.001	< 0.001	< 0.001	< 0.001	< 0.001	< 0.001
CNS	0.779<br>0.529 – 1.146<br>0.204	2.838<br>1.984 – 4.058<br>< 0.001	3.688<br>2.600 – 5.230<br>< 0.001	1.998<br>1.347 – 2.965<br>0.001	2.817<br>1.990 – 3.986<br>< 0.001	1.747<br>1.161 – 2.629<br>0.007	1.781<br>1.164 – 2.726<br>0.008	1.049<br>0.706 – 1.559<br>0.812	1.830<br>1.267 – 2.644<br>0.001
Endocrine	0.747<br>0.457 – 1.220<br>0.243	1.859<br>1.205 – 2.869<br>0.005	2.229<br>1.464 – 3.396<br>< 0.001	0.923<br>0.499 – 1.706<br>0.797	1.399<br>0.872 – 2.244<br>0.164	2.058<br>1.203 – 3.521<br>0.008	1.496<br>0.894 – 2.503<br>0.126	1.405<br>0.844 – 2.341<br>0.191	1.097<br>0.706 – 1.704<br>0.680
Gastrointestinal	2.280<br>1.964 – 2.647<br>< 0.001	1.996<br>1.727 – 2.307<br>< 0.001	2.404<br>2.062 – 2.803<br>< 0.001	4.126<br>3.462 – 4.917<br>< 0.001	5.254<br>4.488 – 6.151<br>< 0.001	2.406<br>2.008 – 2.883<br>< 0.001	1.497<br>1.253 – 1.789<br>< 0.001	1.116<br>0.944 – 1.318<br>0.198	1.808<br>1.563 – 2.092<br>< 0.001
Genitourinary	0.741<br>0.616 – 0.891<br>0.001	0.780<br>0.660 – 0.921<br>0.004	0.955<br>0.798 – 1.144<br>0.619	0.926<br>0.730 – 1.176<br>0.529	0.976<br>0.804 – 1.185<br>0.805	1.346<br>1.087 – 1.665<br>0.006	0.698<br>0.565 – 0.863<br>0.001	0.538<br>0.442 – 0.655<br>< 0.001	0.549<br>0.461 – 0.653<br>< 0.001
Gynecology	0.863<br>0.710 – 1.049<br>0.140	0.853<br>0.711 – 1.024<br>0.089	0.789<br>0.648 – 0.960<br>0.018	1.352<br>1.070 – 1.709<br>0.012	1.121<br>0.911 – 1.379<br>0.282	0.884<br>0.693 – 1.128<br>0.322	0.771<br>0.613 – 0.970<br>0.026	0.852<br>0.688 – 1.055<br>0.141	0.856<br>0.713 – 1.027<br>0.094
Head and Neck	3.158<br>2.490 – 4.005<br>< 0.001	1.723<br>1.362 – 2.179<br>< 0.001	2.588<br>2.014 – 3.326<br>< 0.001	2.987<br>2.321 – 3.844<br>< 0.001	6.512<br>5.166 – 8.210<br>< 0.001	1.392<br>1.027 – 1.885<br>0.033	1.401<br>1.041 – 1.884<br>0.026	1.113<br>0.840 – 1.475<br>0.457	1.387<br>1.091 – 1.763<br>0.008
Hematology	1.254<br>1.059 – 1.485<br>0.009	1.401<br>1.198 – 1.638<br>< 0.001	1.540<br>1.303 – 1.820<br>< 0.001	1.764<br>1.444 – 2.155<br>< 0.001	2.301<br>1.941 – 2.727<br>< 0.001	2.404<br>1.973 – 2.930<br>< 0.001	0.873<br>0.713 – 1.068<br>0.187	0.777<br>0.644 – 0.937<br>0.008	1.200<br>1.022 – 1.411<br>0.026
Intrathoracic	3.190<br>2.681 – 3.796<br>< 0.001	3.600<br>3.023 – 4.288<br>< 0.001	3.667<br>3.063 – 4.389<br>< 0.001	3.852<br>3.145 – 4.719<br>< 0.001	6.670<br>5.565 – 7.993<br>< 0.001	19.364<br>15.508 – 24.180<br>< 0.001	3.112<br>2.522 – 3.838<br>< 0.001	1.994<br>1.640 – 2.424<br>< 0.001	2.925<br>2.470 – 3.463<br>< 0.001
Melanoma	0.544<br>0.380 – 0.780<br>0.001	0.464<br>0.333 – 0.648<br>< 0.001	0.594<br>0.410 – 0.860<br>0.006	0.428<br>0.263 – 0.696<br>0.001	0.561<br>0.373 – 0.845<br>0.006	0.666<br>0.423 – 1.051<br>0.081	0.801<br>0.545 – 1.178<br>0.260	0.892<br>0.626 – 1.270<br>0.525	0.522<br>0.375 – 0.727<br>< 0.001

Covariate	Pain	Tiredness	Drowsiness	Nausea	Appetite	Breath	Depression	Anxiety	Wellbeing
	1.643	1.713	2.050	2.452	2.265	1.832	2.232	1.530	1.390
Sarcoma	1.082 – 2.495	1.131 – 2.596	1.318 – 3.188	1.533 – 3.921	1.448 – 3.545	1.130 – 2.969	1.372 – 3.631	0.962 – 2.434	0.904 – 2.136
	0.020	0.011	0.001	< 0.001	< 0.001	0.014	0.001	0.073	0.133
	8.976	6.634	5.367	14.181	16.916	8.939	3.211	3.338	3.625
Other	3.803 – 21.186	2.513 – 17.511	2.381 – 12.099	6.217 – 32.346	7.626 – 37.521	3.422 – 23.351	1.250 – 8.254	1.351 – 8.244	1.579 – 8.322
	< 0.001	< 0.001	< 0.001	< 0.001	< 0.001	< 0.001	0.015	0.009	0.002

[a] Reported data: Odds ratio estimate (top); 95% CL (middle); p-value (bottom).

[b] Indicates covariate is modelled as a continuous variable.

[c] Indicates covariate is modelling as a categorical variable, with the reference level indicated. P-values in these rows correspond to overall significance of covariates in the modelling
